# Long-term recreational exercise patterns in adolescents and young adults: Trajectory predictors and associations with health, mental-health, and educational outcomes

**DOI:** 10.1371/journal.pone.0284660

**Published:** 2024-03-21

**Authors:** Julie Ayliffe Morgan, Jana Maria Bednarz, Ronnie Semo, Scott Richard Clark, Klaus Oliver Schubert

**Affiliations:** 1 Discipline of Psychiatry, Adelaide Medical School, University of Adelaide, Adelaide, SA, Australia; 2 Adelaide Health Technology Assessment, School of Public Health, University of Adelaide, Adelaide, SA, Australia; 3 National Centre for Vocational Education Research (NCVER), Adelaide, SA, Australia; 4 The Queen Elizabeth Hospital, Central Adelaide Local Health Network, Adelaide, SA, Australia; 5 Northern Adelaide Mental Health Service, SA Health, Salisbury, SA, Australia; 6 headspace Early Psychosis, headspace Adelaide, Sonder, Adelaide, SA, Australia; University of Montenegro: Univerzitet Crne Gore, MONTENEGRO

## Abstract

Individual and societal factors influencing the formation of long-term recreational exercise habits during the transition from adolescence to young adulthood are not well explored. Using data from the Longitudinal Survey of Australian Youth (LSAY), a population-representative cohort study of Young People followed from age 15 to 25, we aimed to (1) model longitudinal recreational exercise trajectories from age 16 to 24, (2) examine predictors at age 15 of entering these trajectories, and (3) explore the association between the trajectories and health, mental health and educational achievement outcomes measured at the final study wave (age 25). Self-reported recreational exercise frequency data from 9353 LSAY participants were analysed using group-based trajectory modelling. We modelled the evolution of two patterns of recreational exercise behaviour: *daily exercise*, as per public health guidelines (Model 1); and *at least once weekly exercise* (Model 2). Model 1 trajectories were *guideline-adherent exercisers* (17.9% of the sample), *never guideline exercisers* (27.5%), *guideline drop-outs* (15.2%) and *towards guideline* (39.4%); Model 2 trajectories were *weekly exercise* (69.5% of the sample), *decreasing* (17.4%), *increasing* (4.8%), and *infrequent* (8.3%). For both models, at age 15, trajectory membership was predicted by gender, self-efficacy, time spent participating in sport, time spent watching TV, parental socioeconomic status, and academic literacy. At age 25, people in the *guideline-adherent exerciser* trajectory (model 1) reported better general health relative to other trajectories, Those in the *weekly exerciser* trajectory (model 2) had better general health and reduced rates of psychological distress, were happier with life and were more optimistic for the future relative to participants in *less than weekly* trajectory groups. Exercise-promoting interventions for Young People should specifically address the needs of females, people with low self-efficacy, reluctant exercisers, higher academic achievers, and those experiencing socioeconomic disadvantage.

## 1. Introduction

The transition from adolescence to young adulthood is a critical time for the development of behaviours that influence health and wellbeing across the life span. Many chronic mental and physical health conditions manifest for the first time during this stage, including depression, premature diabetes, and cardiovascular disease [1–3]. Promoting positive health behaviours in young people can have benefits during youth, reduce the burden of disease in adulthood, and indirectly improve health in the next generation of children [4]. While population-level interventions for young people may powerfully impact social and economic outcomes [5], longitudinal data about the development of health behaviours in 15 to 25-year-olds remain relatively sparse, prompting calls for global initiatives to improve behavioural health research in this age group [4, 6–8].

Physical exercise, being a repetitive activity to maintain or improve fitness [9], can confer long-term physical- and mental health benefits. In cross-sectional studies of adult populations, positive associations between exercise and cardiometabolic health, mental health, and quality of life have been reported [10, 11]. Similarly, in young people, cross-sectional studies have shown that physical exercise is associated with reduced risk for psychological distress [12], and with higher levels of bone health [13], self-esteem [12], flourishing [14], and health-related quality of life [15]. While some studies have demonstrated beneficial effects of cross-sectionally assessed exercise in youth on later adult outcomes [16, 17], distinct exercise patterns or *trajectories* during the transition from adolescence to young adulthood have received little attention [18]. Group-based trajectory modelling (GBTM) allows exploration of heterogeneity in behavioural trends over time by identifying distinct latent subgroups within a population. Predictors of latent group membership can be examined, and latent group membership itself may be used in turn to explain differences in particular outcomes. By identifying predictive factors for particular trajectory groups, these approaches have the potential to inform health- and educational policies and aid in the targeting of preventive measures in high risk populations [19].

Here, we used data from the 2006 Longitudinal Survey of Australian Youth (LSAY), a population-representative cohort study of Young People to inform health- and educational policy. Participants were followed from age 15 to 25, with annual assessments from 2006–2016. Self-reported recreational exercise frequency, outside school or work, was assessed on five occasions over 8 years, between ages 16 and 24. We aimed to (1) model longitudinal recreational exercise trajectories from age 16 to 24, (2) examine predictors at age 15 of entering these trajectories, and (3) explore the association between the trajectories and health, mental health and educational achievement outcomes measured at the final study wave (age 25).

## 2. Materials and methods

### 2.1 Data source and participants

Participants were from The Longitudinal Survey of Australian Youth (LSAY) [20]. LSAY is managed by the Australian Council for Educational Research (ACER) and the Commonwealth Department of Education, Science and Training (DEST) and is designed to provide policy-relevant information about the transition from school to work for youth. Data were collected annually from 2006 at age 15 until participants turned 25 and were deposited within the Australian Data Archive (ADA) at the Australian National University. The study is jointly managed by the Australian Government Department of Education, Skills and Employment and the National Centre for Vocational Education Research (NCVER) and adheres to guidelines set by the National Health and Medical Research Council’s National Statement on the Ethical Conduct of Human Research. Ethics approval for the LSAY survey was granted by the Australian Institute of Family Studies Ethics Committee [21]. The LSAY data are publicly available after an online registration and application process (www.ada.edu.au/). Approval was granted by The Australian Data Archive for controlled access to the LSAY 2015 cohort data (Version 1.0), and subsequently deidentified, non-reidentifiable data were available for analysis.

Data were selected from the 2006 LSAY cohort which recruited 14, 170 students, at the age of 15, from 356 Australian schools involved in the Program for International Student Assessment (PISA). Participant consent was gained both in writing and verbally for adult participants. For participants under the age of eighteen, following the PISA assessment, parents were sent information about LSAY and the opportunity to opt out for their child. All participants were first assessed in 2006, followed up in 2007 by telephone, and then interviewed annually by computer assisted telephone interviews. From 2012, participants responded via an online portal until the completion of the survey in 2016.

Recreational exercise engagement was self-reported in 2007, 2008, 2009, 2011, and 2014 [20]. Longitudinal modelling was based on 9,535 participants with exercise data available. Health, mental health, and vocational outcomes were recorded in wave 10 in 2016, when participants were aged between 25 and 26 years [20].

We specified all exposure-, predictor-, and outcome variables *a priori*, based on literature review. Recreational exercise engagement was measured on a 7-point Likert-type scale, in response to the survey question “Outside study or work, how often do you play sport or do regular exercise?” (1 = every day, 2 = at least once a week (but not every day), 3 = at least once a month but less than once a week, 4 = at least once every 3 months but less often than once a month, 5 = at least once a year but less often than once every 3 months, 6 = less often than once a year, 7 = Never) [20]. Given the intervals between response categories were unequal, we formed two binary variables describing level of exercise participation, which were used in subsequent group-based trajectory modelling. The first definition defined exercise participation according to adherence to international guidelines [22] ‐ the *daily guideline-adherent* exercise versus *non-guideline adherent* exercise (model 1) with categories of daily exercise (daily ‘guideline-adherent’ exercise) and non-guideline exercise (all other response categories; ‘non-guideline’). However, because many young people and adults do not meet the international exercise guidelines, we were also investigated recreational exercise engagement that did not conform to international guidelines. The second definition of exercise participation for model 2 differentiated between low levels of exercise participation (any response describing less than once-weekly participation; ‘infrequent’ exercise) and more regular engagement (at least once a week but not every day; ‘weekly’ exercise), and was the *non-guideline adherent weekly exerciser* trajectory (model 2).

We examined relationships between the exercise trajectories and sociodemographic and other variables we expected to be associated with exercise participation, based on previous research findings. These included age, gender, socio-economic status (maternal and paternal International Socio-Economic Index of Occupational Status (ISEI)); indices of self-worth and self-efficacy; ethnicity (Aboriginal or Torres Strait Islander (ATSI)); attitudes towards sport, time spent playing sport, time spent watching television; and academic literacy and students interest in school [20, 23–27]. Students’ enjoyment of school was also included [20]. These candidate predictors were assessed in 2006 at age 15/16, prior to the initial period of the exercise trajectories (see Table 1).

**Table 1:** Candidate predictor variables for trajectory group membership.

Risk factor	LSAY item	Year measured	Unit/values (range)	Proportion of data missing (as % of N = 9353)
Sex	Gender	2006	(1) Male	0 (no missing data)
			(2) Female	
International Socio-Economic Index of Occupational Status (proxy for SES)	Father’s ISES score	2006	1–100	7.45%
	Mother’s ISES score	2006	1–100	11.1%
Self-efficacy	“Compared with most students in your year level, how well are you doing in your subjects overall?”	2006	(1) Below average	0.77%
			(2) About average	
			(3) Above average	
Self-worth	“My school is a place where I know I can do well enough to be successful”	2006	(1) Agree	0.71%
			(2) Disagree	
	“I am a success as a student”	2006	(1) Agree	1.28%
			(2) Disagree	
	“My school is a place where teachers give me marks I deserve”	2006	(1) Agree	0.97%
			(2) Disagree	
Ethnicity	ATSI-status	2006	(1) Indigenous	0 (no missing data)
			(2) Non-indigenous	
Sports participation	On average, how many hours per week do you spend playing sport?	2006	minutes per week	2.11%
Screen time: TV	On average, how many hours do you spend each week on watching TV?	2006	minutes per week	1.85%
Enjoyment of school	My school is a place where I get enjoyment from being here	2006	(1) Agree	0.92%
			(2) Disagree	
Academic aptitude	Plausible value in maths	2006	670–3950	0 (no missing data)
	Plausible value in reading	2006	670–3950	0 (no missing data)
	Plausible value in science	2006	670–3950	0 (no missing data)
	Academic literacy (sum of maths, reading and science scores)	2006	2010–11850	0 (no missing data)
Work at school	My school is a place where: I am given the chance to do work that really interests me	2006	(1) Agree	0.74%
			(2) Disagree	

Note: Maximum plausible upper limits were imposed for sports participation (responses >40 hours/week excluded, n = 91) and TV time (responses > 74 hours/week excluded n = 42).

The exercise trajectories for Model 1 (*daily guideline-adherent* exercise versus *non-guideline adherent* exercise) and Model 2 (*non-guideline adherent weekly exerciser* trajectory) were tested for associations with several self-reported indicators of mental health, physical health, and vocational attainment were measured in 2016 (at age 25). These included the Kessler-6 psychological distress score (K6), a validated measure widely used as an index of mental health in Australian population-based studies where comprehensive assessments are not feasible [20, 28]. Other measures taken at this timepoint included a single item from the SF-36 to qualify general health (“How would you say your health is?”) [20, 29], and items regarding on satisfaction with life and view of one’s future, completion of high school, completion of a post school qualification, and participation in the labour force [20] (see Table 2). K6 scores were dichotomised into either 0 = lower risk of mental illness (scores from 6–18) or 1 = greater risk of mental illness (scores > 19) [30]. Predictor variables of exercise trajectory groups were included in outcome analyses to control for potential confounding.

**Table 2:** Outcome variables assessed at age 25.

Outcome	LSAY item	Year measured	Unit/values (range)
General health	“In general, how would you say your health is?” (from SF-36)	2016	(1) excellent
			(2) very good
			(3) fair
			(4) poor
Greater risk of mental illness	Kessler-6 (K6) questionnaire	2016	(1) lower risk of mental illness (score of 8–18)
			(2) greater risk of mental illness (score of 19–30)
Life satisfaction	“How happy are you with your life as a whole?”	2016	(1) very happy or happy
			(2) unhappy or very unhappy
View of one’s future	“How happy are you with your future?”	2016	(1) very happy or happy
			(2) unhappy or very unhappy
Completion of high school	Completed Year 12 or Certificate II or higher	2016	(1) Year 12 completed
			(2) Year 12 not completed
Completion of post-school qualification	Completed any post-school qualification	2016	(1) Qualification completed
			(2) Qualification not completed
Participation in the labour force	Derived variable	2016	(1) Participating in labour force (employed)
			(2) Not participating in labour force (unemployed)

### 2.2 Statistical methods

We described trajectories of daily (guideline-adherent) exercise (Model 1) and weekly exercise (Model 2) from adolescence to young adulthood using group-based trajectory modelling (GBTM) procedures. GBTM is a semi-parametric technique that identifies subgroups of individuals who follow similar patterns of a behaviour over time [30]. We assumed a Bernoulli distribution since the measures of exercise behaviour (daily/guideline-adherent and weekly) were binary. GBTM uses maximum likelihood to handle missing data under an assumption of missing at random. All survey participants with self-reported exercise data available at least one time point were included (N = 9353) in the trajectory modelling.

For each trajectory model of daily and weekly exercise, we determined the optimal number of trajectory groups and the shape (intercept, linear, quadratic, cubic) of each trajectory based on the Bayesian Information Criterion which measures goodness of fit [31]. Trajectory groups containing less than 1% of the sample were excluded. Statistical analyses were performed in Stata (version 15 StataCorp Texas) with the level of statistical significance set to 0.05. The ‘tra’j add-on for Stata was utilised to determine the optimal number of trajectory groups suggested by the data, and their shape (polynomial-order) [32] through a series of models with one to four groups of varying polynomial order. There was little evidence against the null hypothesis that the exercise data were Missing Completely At Random (MCAR) from Little’s test (χ_(33)_^2^ = 20.2, p = 0.961), so the assumption that attrition is independent of trajectory group is reasonable. After the optimum number of trajectories and their shapes were determined, participants were then assigned to the trajectory group for which they had the highest probability for membership. This was performed separately for daily exercise and weekly exercise. We then investigated the pre-specified baseline factors for their association with trajectory group membership using multinomial logistic regression. All predictor variables had low amounts of missing data (<5%).

We assessed the association between the trajectories of daily and weekly exercise behaviour and outcomes measured at age 25–26 for the subset of participants with outcome data available. We used logistic regression for categorical outcomes, and linear regression for continuous outcomes and adjusted for potential confounders (gender, indigeneity, student socio-economic status (SSES), father ISEI, self-efficacy, academic achievement, and sport participation, TV time, and enjoyment of school at baseline in 2006) [20]. Standard errors were adjusted to account for clustering of participants by school. Goodness of fit was evaluated using the Hosmer-Lemeshow test for logistic regression models, and by visual inspection of residual plots for normality and homoscedasticity for linear regression models.

## 3. Results

### 3.1 Patterns of longitudinal recreational exercise participation between ages 16 and 24

**Fig 1:**
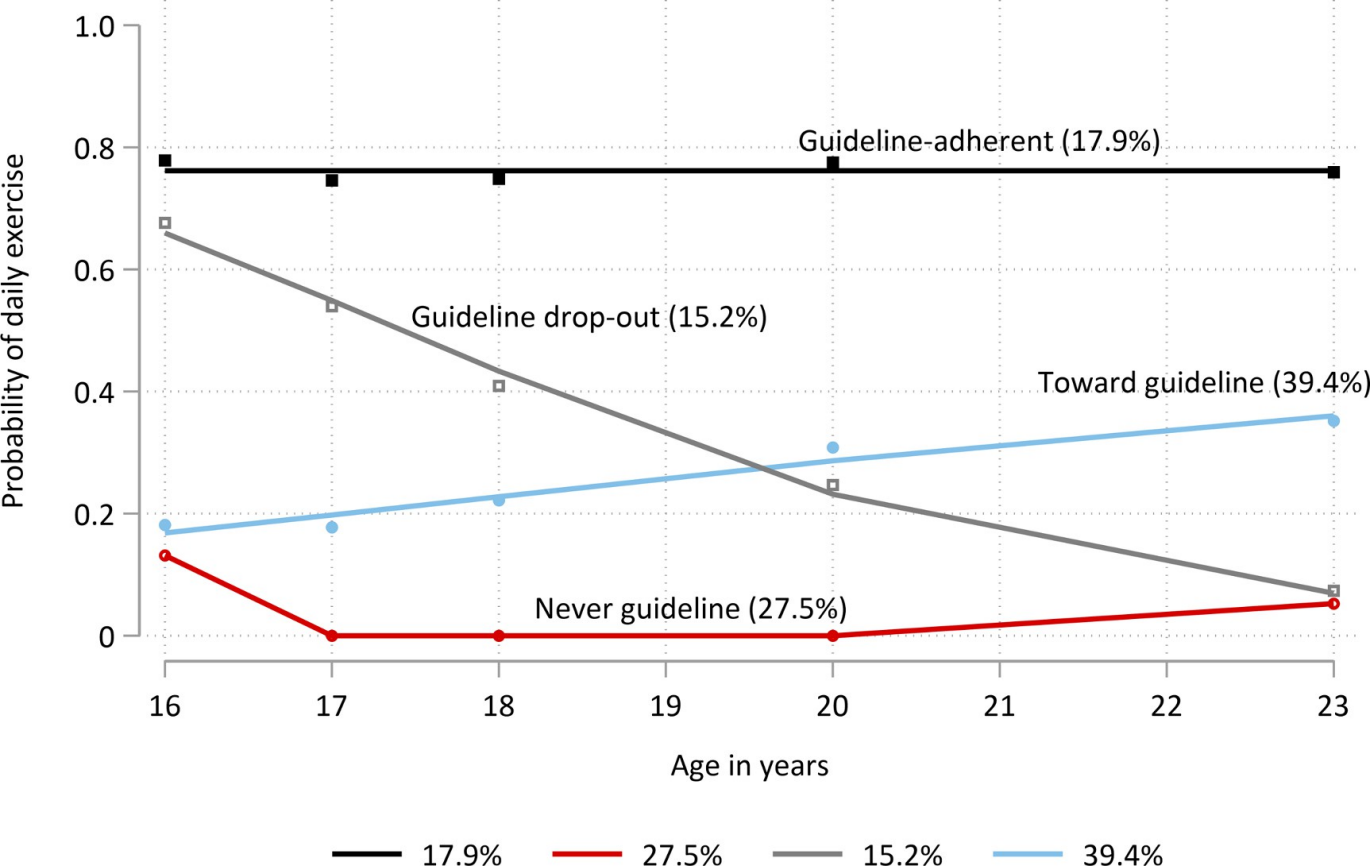
Models for 8-year physical exercise trajectories from age 16 to 24, exercise trajectory model 1. Of the 9,353 young people included in the model, 17.9% had a high probability of consistently meeting the WHO guideline recommendation of daily exercise, over 8 years (*guideline*, black line). 27.5% were unlikely to ever meet recommendations (*never guideline*, dark red line). 15.2% of the sample were initially meeting daily exercise requirements but dropped out over time (*guideline drop-outs*, grey line). 39.4% of young people had a low initial probability of meeting guideline requirements but were more likely as time progressed (*towards guideline*, blue line).

Exercise trajectory model 1 evaluated the recreational exercise patterns of *guideline* vs *non-guideline* exercisers from age 16 to 24. (Proportions of young people in these categories per time point are shown in S1 Fig. see Supplementary Information). Trajectory model 1 was a four-group model (with groups of polynomial order 0, 0, 2 and 2, respectively) (Fig 1) and all trajectory subgroups included more than 1% of the sample. The two groups showing stable probabilities of exercise over time were labelled *guideline* (a high probability of daily exercise over time; 17.9% of the sample) and *never guideline* (low probability of daily exercise; 27.5% of the sample). The two groups with changes in probability over time were labelled *guideline drop-outs* (15.2% of the sample) and *towards guideline* (39.4% of the sample).

**Fig 2:**
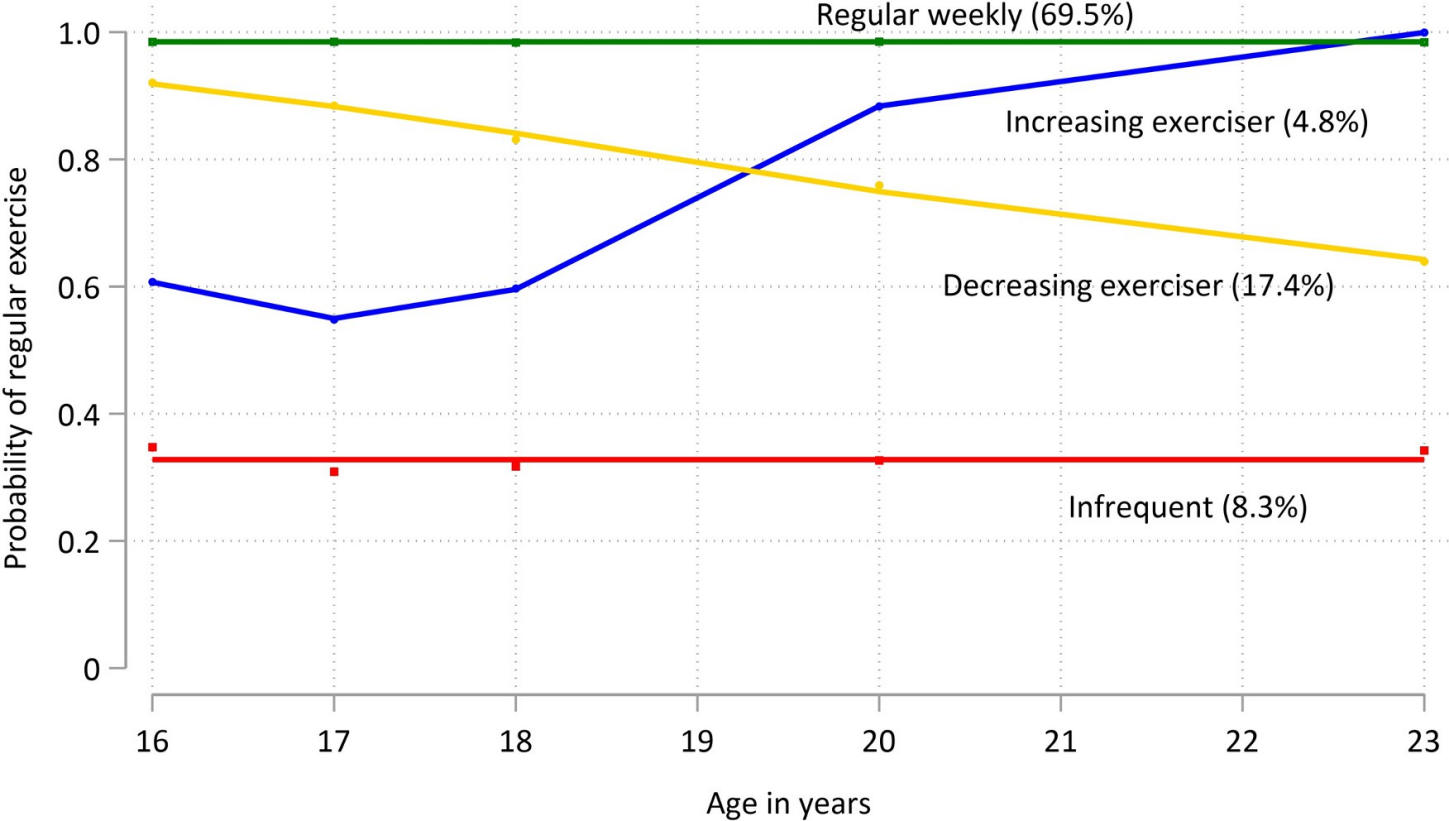
Model for 8-year physical exercise trajectories from age 16 to 24, exercise trajectory model 2. 8.3% of young people had a persistently low probability of meaningful recreational physical exercise engagement, reporting less than one occasion per week (*infrequent*, red line). In contrast, 69.5% had a high probability of engaging consistently in more regular exercise (*weekly*, green line). 17.4% of the sample had an initially high probability of regular exercise that declined over time (*decreasing exerciser*, yellow line). 4.8% had a modest initial probability of regular exercise that increased from age 17 (*increasing-exerciser*, blue line).

Exercise in trajectory model 2 used the binary variable *infrequent* vs *weekly* exercisers. (Proportions of young people in these categories at each assessment time point are shown in S2 Fig (see Supplementary Information). Model 2 was also a four-group model (with groups of polynomial order 0, 0, 2 and 2, respectively) (Fig 2), and again all exercise trajectory subgroups including more than 1% of the sample. We labelled the two stable groups *infrequent* exercisers (with high probability of very little exercise over time, 8.3% of the sample) and *weekly* exercisers (exercising at least once weekly but not daily ‐ 69.5% of the sample). The two groups showing change in behaviour over time were labelled *declining-exercisers* (17.4%), and *increasing-exercisers* (4.8%), respectively.

### 3.2 Predictors of long-term adherence to exercise guidelines

**Fig 3:**
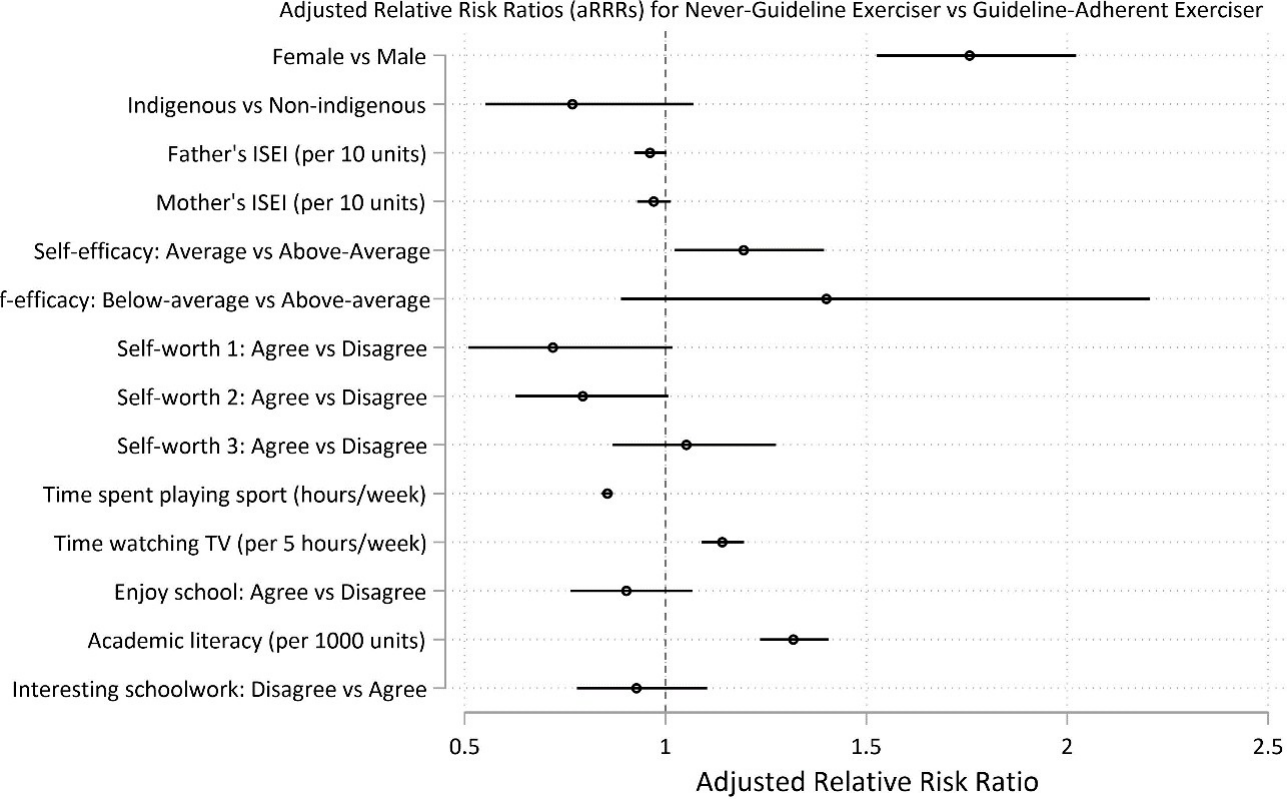
Predictors at age 15 for long-term recreational physical exercise participation between the ages of 16 and 24. Predictors for guideline-adherent (= daily) exercise. Predictors are shown as adjusted Relative Risk Ratios (aRRRs) for falling into the *never guideline* exerciser trajectory (Fig 3), the *towards guideline* trajectory (Fig 4) and the *guideline drop-out* trajectory (Fig 5), compared to the risk of following the *guideline* exercise trajectory.

**Fig 4:**
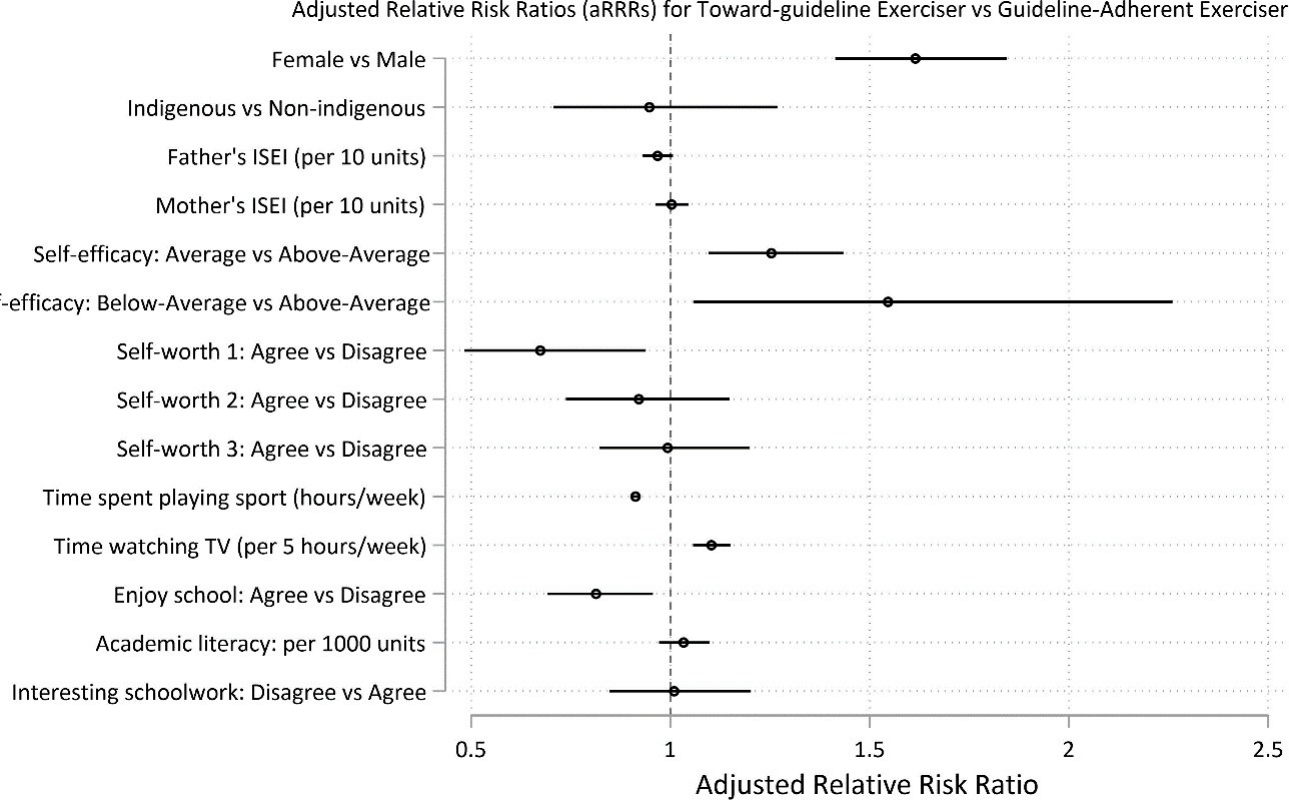
Predictors at age 15 for long-term recreational physical exercise participation between the ages of 16 and 24. Predictors for guideline-adherent (= daily) exercise. Predictors are shown as adjusted Relative Risk Ratios (aRRRs) for falling into the *never guideline* exerciser trajectory (Fig 3), the *towards guideline* trajectory (Fig 4) and the *guideline drop-out* trajectory (Fig 5), compared to the risk of following the *guideline* exercise trajectory.

**Fig 5:**
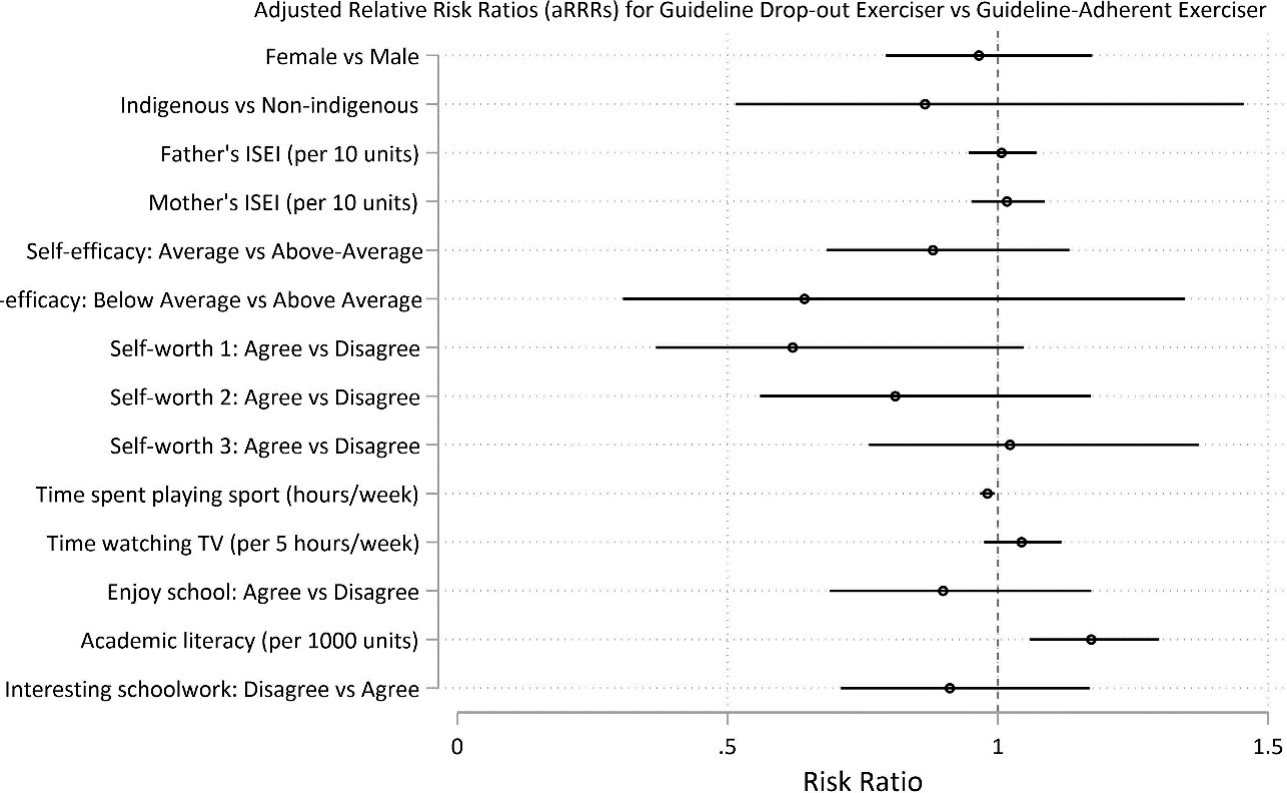
Predictors at age 15 for long-term recreational physical exercise participation between the ages of 16 and 24. Predictors for guideline-adherent (= daily) exercise. Predictors are shown as adjusted Relative Risk Ratios (aRRRs) for falling into the *never guideline* exerciser trajectory (Fig 3), the *towards guideline* trajectory (Fig 4) and the *guideline drop-out* trajectory (Fig 5), compared to the risk of following the *guideline* exercise trajectory.

Predictors of long-term adherence to daily exercise (and thus to exercise guidelines) at age 15 were the factors that reduced the risk of membership in a trajectory group other than daily exercise. Predictor factors are reported as the adjusted Relative Risk Ratio (aRRR) for membership to a non-guideline group vs the guideline-adherent group (95% CI; p value). More time playing sport was a strong predictor of long-term maintenance of daily exercise: aRRR for each hour playing sport per week for *never guideline* vs *guideline* = 0.86, 95% CI 0.84, 0.87; p < .0001; for *guideline dropout* vs *guideline* = 0.98, 95% CI 0.97, 0.99; p<0.01; for *towards guideline* vs *guideline* = 0.91 95% CI 0.90, 0.92; p<0.001 (Figs 3–5 and S1 Table).

Conversely, there were multiple factors that increased risk of membership in a group for less than daily exercise (reported as aRRR for membership to a non-guideline vs guideline-adherent group, 95% CI; p). These were: female gender: aRRR for *never guideline* vs *guideline* = 1.76, 95% CI 1.53, 2.02; p < .0001; for *towards guideline* vs *guideline* = 1.61, 95% CI 1.41, 1.84; p = 0.001), lower academic self-efficacy (relative to higher self-efficacy): aRRR for *never guideline* vs *guideline* = 1.20, 95% CI 1.02, 1.39; p = 0.025; for *towards guideline* vs *guideline* = 1.25, 95% CI 1.09, 1.43; p<0.001), greater academic literacy: aRRR per 1000 point increase for *never guideline* vs *guideline* = 1.32, 95% CI 1.24, 1.40; p<0.001; *guideline dropout* vs *guideline* = 1.17, 95% CI 1.06, 1.30; p = 0. and more time watching TV: aRRR per 5 hour increase per week for *never guideline* vs *guideline* = 1.14, 95% CI 1.09, 1.20; p < .0001; *towards guideline* vs *guideline* = 1.10 95% CI 1.05, 1.15; p<0.001) (Figs 3–5 and S1 and S2 Tables).

### 3.3 Risk factors for insufficient long-term recreational exercise

**Fig 6:**
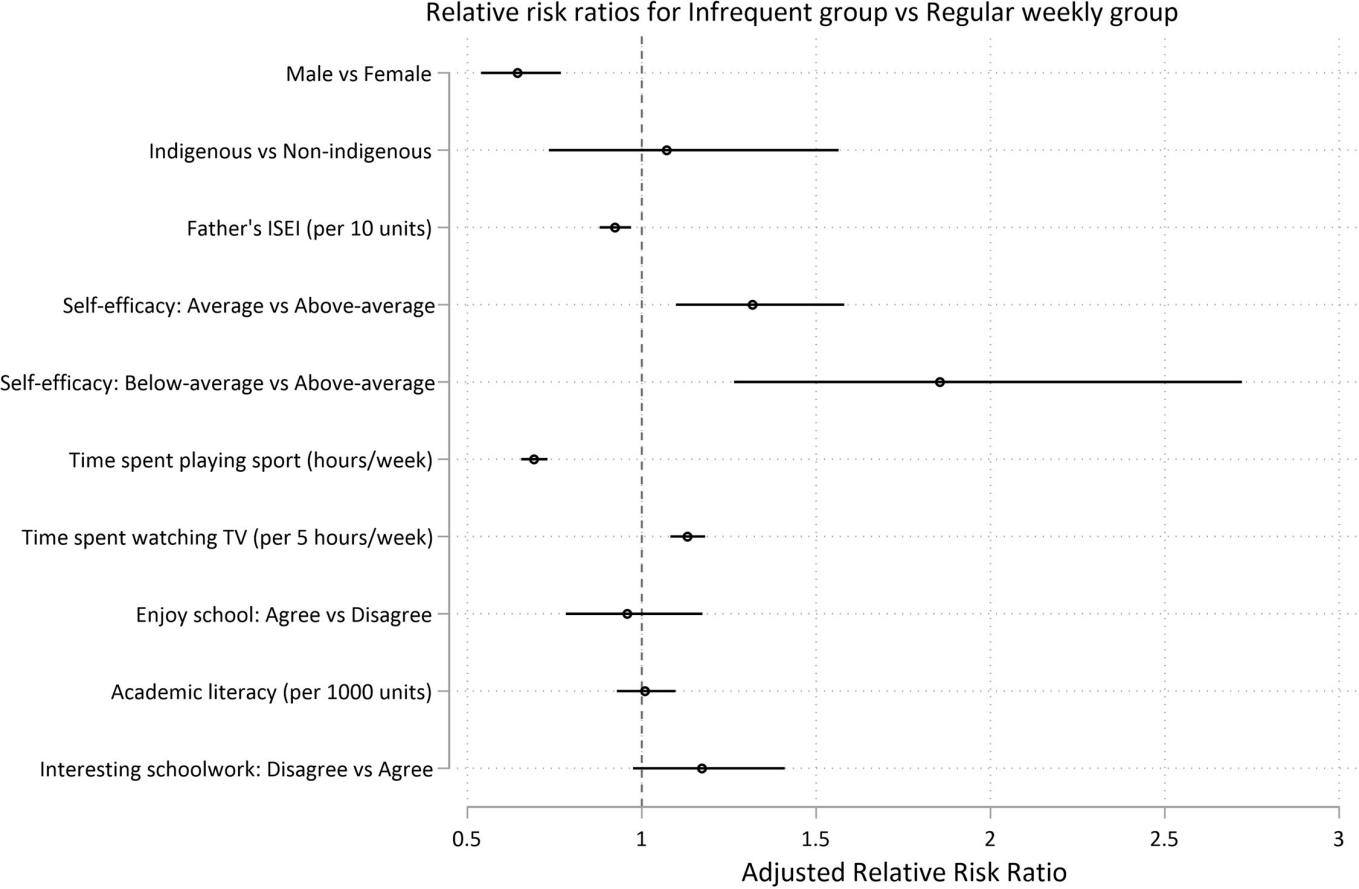
Predictors at age 15 for long-term recreational physical exercise participation between the ages of 16 and 24. Risks for insufficient (*less than weekly*) recreational exercise (Model 2). Risks are shown as adjusted Relative Risk Ratios (aRRRs) for falling into the infrequent exerciser trajectory (Fig 6), the decreasing exerciser trajectory (Fig 7) and the increasing exerciser trajectory (Fig 8), compared to the risk of following the *weekly* exercise trajectory. ISEI: International Socio-Economic Index of Occupational Status.

**Fig 7:**
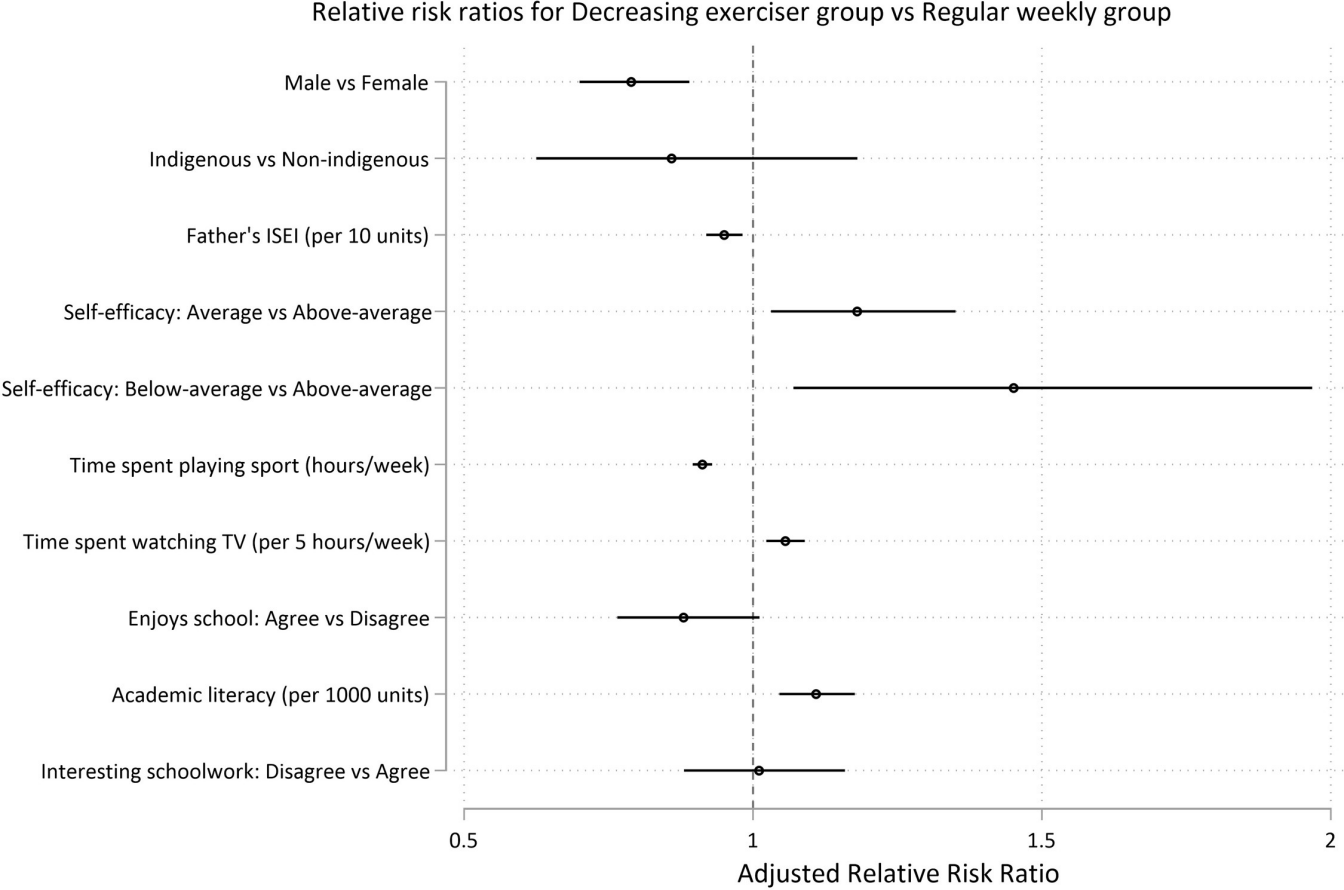
Predictors at age 15 for long-term recreational physical exercise participation between the ages of 16 and 24. Risks for insufficient (*less than weekly*) recreational exercise (Model 2). Risks are shown as adjusted Relative Risk Ratios (aRRRs) for falling into the infrequent exerciser trajectory (Fig 6), the decreasing exerciser trajectory (Fig 7) and the increasing exerciser trajectory (Fig 8), compared to the risk of following the *weekly* exercise trajectory. ISEI: International Socio-Economic Index of Occupational Status.

**Fig 8:**
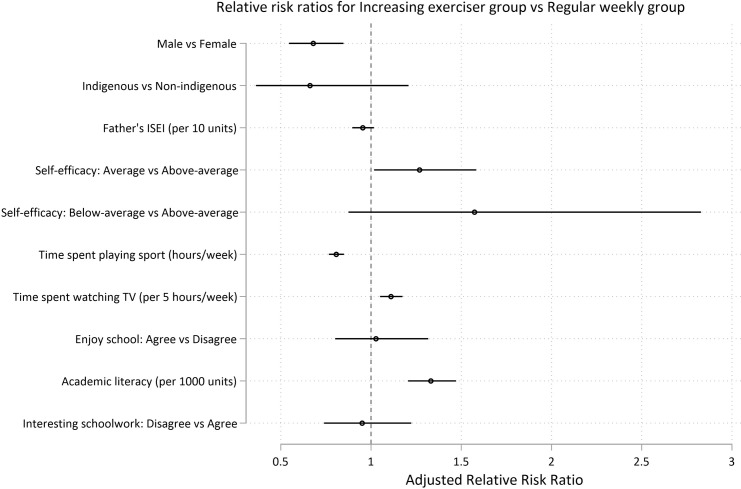
Predictors at age 15 for long-term recreational physical exercise participation between the ages of 16 and 24. Risks for insufficient (*less than weekly*) recreational exercise (Model 2). Risks are shown as adjusted Relative Risk Ratios (aRRRs) for falling into the infrequent exerciser trajectory (Fig 6), the decreasing exerciser trajectory (Fig 7) and the increasing exerciser trajectory (Fig 8), compared to the risk of following the *weekly* exercise trajectory. ISEI: International Socio-Economic Index of Occupational Status.

At age 15, the risk factors for membership in a group engaging in consistently less exercise (infrequent exercisers, increasing exercisers, and declining exercisers) relative to the weekly group were as follows: female gender (aRRR for *infrequent exercisers* = 1.55, 95% CI 1.30, 1.85; p < .0001 ; aRRR for *declining exercisers* = 1.27, 95% CI 1.12, 1.43; p < .0001; aRRR for *increasing exercisers* = 1.47, 95% CI 1.18, 1.83; p = 0.001), increased TV time (for every 5 hours of TV per week, aRRR for *infrequent exercisers* = 1.13, 95% CI 1.08, 1.18; p < .0001 ; aRRR for *declining exercisers* = 1.06, 95% CI 1.02, 1.09; p = 0.001; aRRR for *increasing exercisers* = 1.11, 95% CI 1.05, 1.17; p < .0001), and average levels of academic self-efficacy (versus above-average self-efficacy) (aRRR for *infrequent exercisers* = 1.32, 95% CI 1.10, 1.18; p = 0.003; aRRR for *declining exercisers* = 1.18, 95% CI 1.03, 1.35; p = 0.016; aRRR for *increasing exercisers* = 1.27, 95% CI 1.02, 1.58; p = 0.035). Higher sports participation at age 15 reduced the risk of being in the insufficient exercise trajectories (for each hour spent playing sport per week, aRRR for *infrequent exercisers* = 0.69, 95% CI 0.65, 0.73; p < .0001 ; aRRR for *declining exercisers* = 0.91, 95% CI 0.90, 0.93; p < .0001; aRRR for *increasing exercisers* = 0.81, 95% CI 0.77, 0.85; p < .0001) (Figs 6–8 and S3 and S4 Tables).

Relative to the *weekly* exercise group, below average self-efficacy (versus above-average self-efficacy) was a risk factor for membership in *infrequent exercisers* and *declining-exerciser* groups (aRRR for *infrequent exercisers* = 1.85, 95% CI 1.26, 2.72; p = 0.002; aRRR for *declining exercisers* = 1.45, 95% CI 1.07, 1.97; p = 0.017) (Figs 6, and 7). Higher paternal ISEI was protective against membership in the *infrequent exercisers* and *declining-exerciser* groups (for every 10 units in ISEI score, aRRR for *infrequent exercisers* = 0.92; 95% CI 0.88, 0.97; p = 0.001; aRRR for *declining exercisers* = 0.95, 95% CI 0.92, 0.98; p = 0.002) (Figs 6–8 and S3 and S4 Tables).

Young people with higher academic literacy scores were more likely to be in the *decreasing exerciser* or *increasing exerciser* groups relative to the *weekly* exercise group (for every 1000 units, aRRR for *decreasing exerciser* = 1.11; 95% CI 1.05, 1.18; p = 0.001; aRRR for *increasing exerciser* = 1.33, 95% CI 1.20, 1.47; p < .0001) (Figs 7 and 8). Indigenous status, measures of self-worth, enjoyment of school, or interest in schoolwork were not found to be significantly associated with trajectory group (Figs 6–8 and S3 and S4 Tables).

### 3.4 Associations of long-term *daily* exercise with health, mental health, and educational outcomes

Young people who participated in daily recreational exercise between age 16 and 24 reported better general health at age 25 than those who did not exercise daily (i.e, *never guideline*, *guideline dropouts*, and *towards guideline* groups). However no additional advantages on measured indicators of mental health and vocational attainment were identified. This association remained after the adjustment for possible confounders of gender, ethnicity, participants’ ISEI score, and the predictors of trajectory group membership.

**Fig 9:**
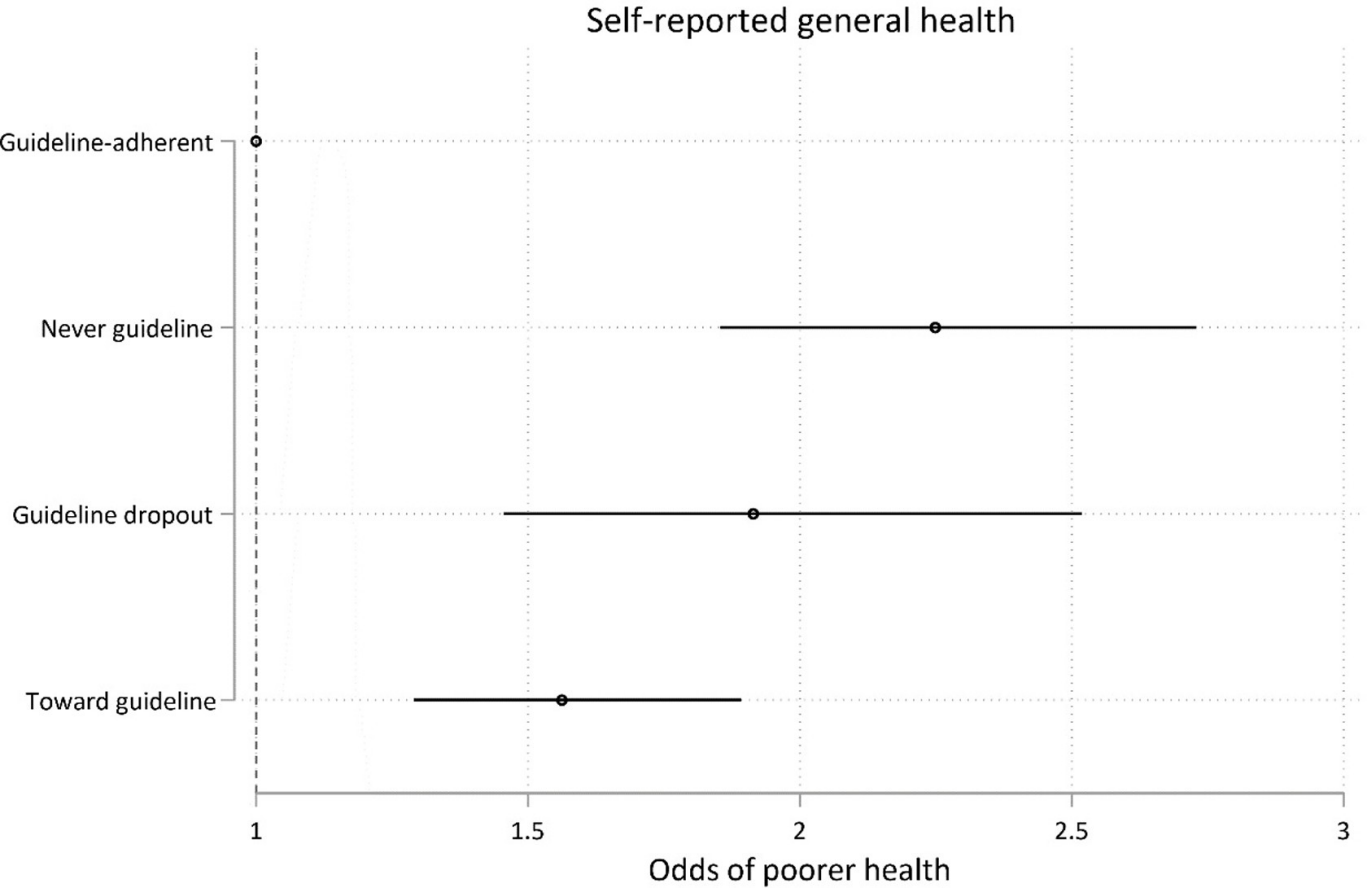
Model 1 trajectory group associations with health-, mental health-, and educational outcomes at age 25. Associations expressed as adjusted odds ratios (ORs) describing the odds of experiencing each outcome of interest for participants who undertake less than daily recreational physical exercise (the *never guideline*, *guideline dropouts*, and *towards guideline* exercise trajectories) vs participants undertaking the recommended level of recreational physical exercise (*guideline* exercisers). Less than *guideline* levels of recreational exercise had poorer self-reported general health (Fig 9).

Compared to the *guideline* adherent daily exercise group, the *never guideline*, *guideline dropouts*, and *towards guideline* groups had significantly higher odds of poorer self-reported general health. Results are reported as adjusted Odds Ratio (aOR) of lower vs higher self-reported general health, 95% CI; p value: *guideline* vs *never guideline* = 2.24, 95% CI 1.85, 2.73; p < .0001; *guideline* vs *guideline dropouts* = 1.91, 95% CI 1.45, 2.51; p < .0001; *guideline* vs *towards guideline* = 1.56, 95% CI 1.29, 1.90; p < .0001) (Fig 9). There were no associations between exercise trajectory groups and mental illness, life satisfaction at age 25 (happiness with the future or happiness with life overall), year 12 completion, the attainment of a post school qualification, or participation in the labour force (see S3–S8 Figs and S5 Table).

### 3.5 Associations of long-term *less-than-weekly* exercise with health, mental health, and educational outcomes

**Fig 10:**
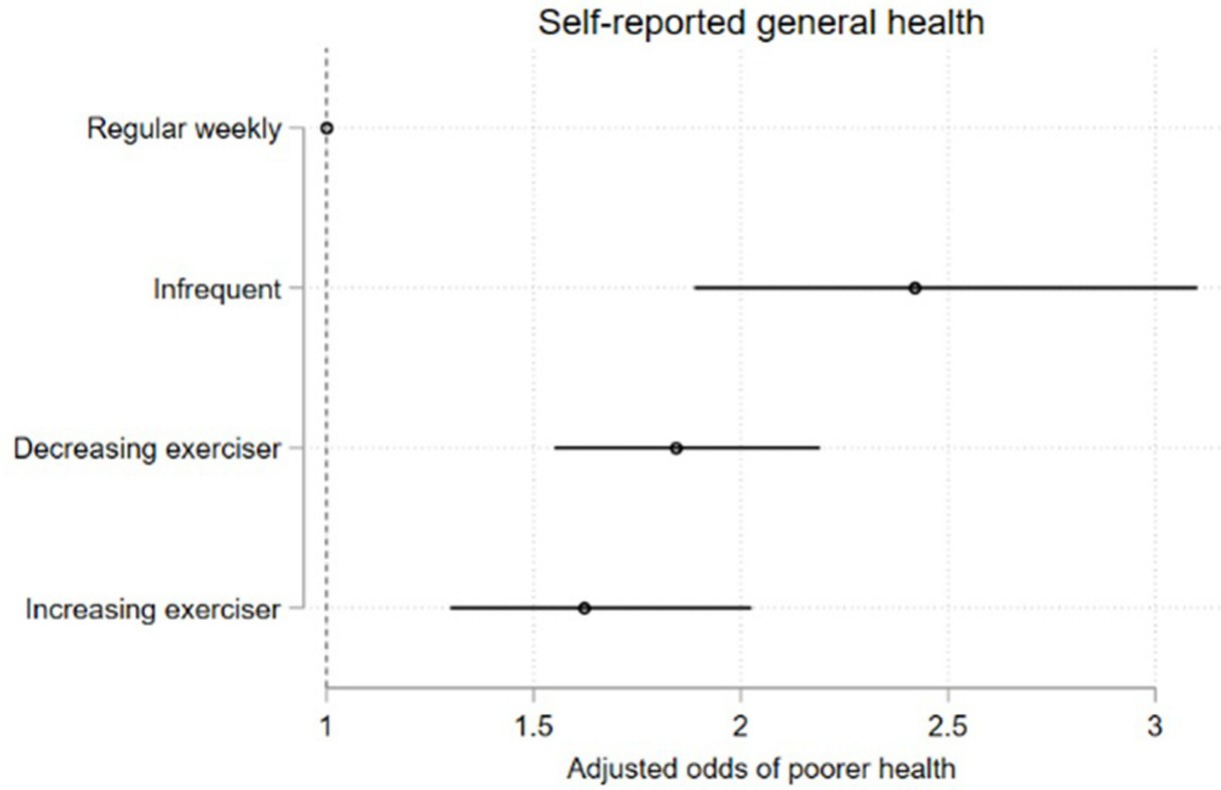
Model 2 trajectory group associations with health-, mental health-, and educational outcomes at age 25. Associations expressed as adjusted odds ratios (aORs) describing the odds of experiencing each outcome of interest for participants who undertake lower levels of recreational physical exercise (infrequent exercisers, decreasing exercisers, increasing exercisers) vs participants undertaking a higher level of recreational physical exercise (*weekly* exercisers). Outcomes are Fig 10) poorer general health; Fig 11) mental illness; Fig 12) overall satisfaction with the future; Fig 13) overall satisfaction with life.

**Fig 11:**
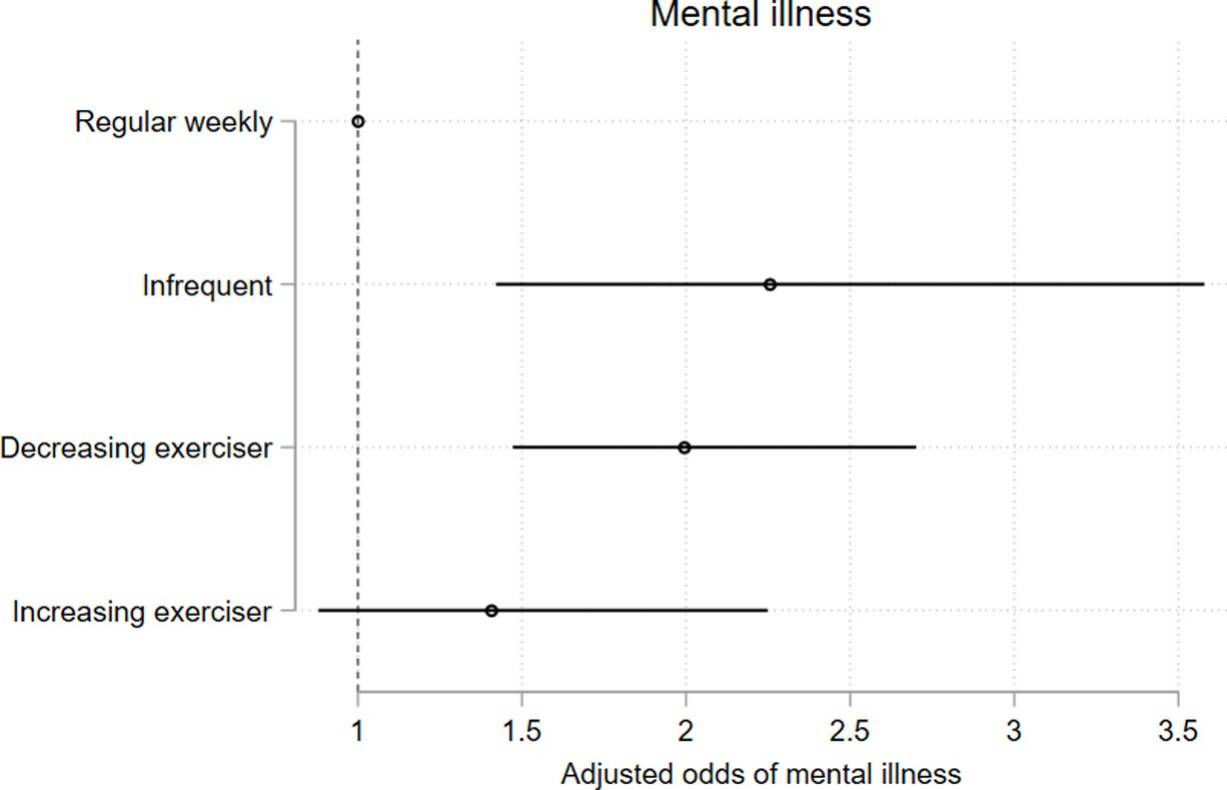
Model 2 trajectory group associations with health-, mental health-, and educational outcomes at age 25. Associations expressed as adjusted odds ratios (aORs) describing the odds of experiencing each outcome of interest for participants who undertake lower levels of recreational physical exercise (infrequent exercisers, decreasing exercisers, increasing exercisers) vs participants undertaking a higher level of recreational physical exercise (*weekly* exercisers). Outcomes are Fig 10) poorer general health; Fig 11) mental illness; Fig 12) overall satisfaction with the future; Fig 13) overall satisfaction with life.

**Fig 12:**
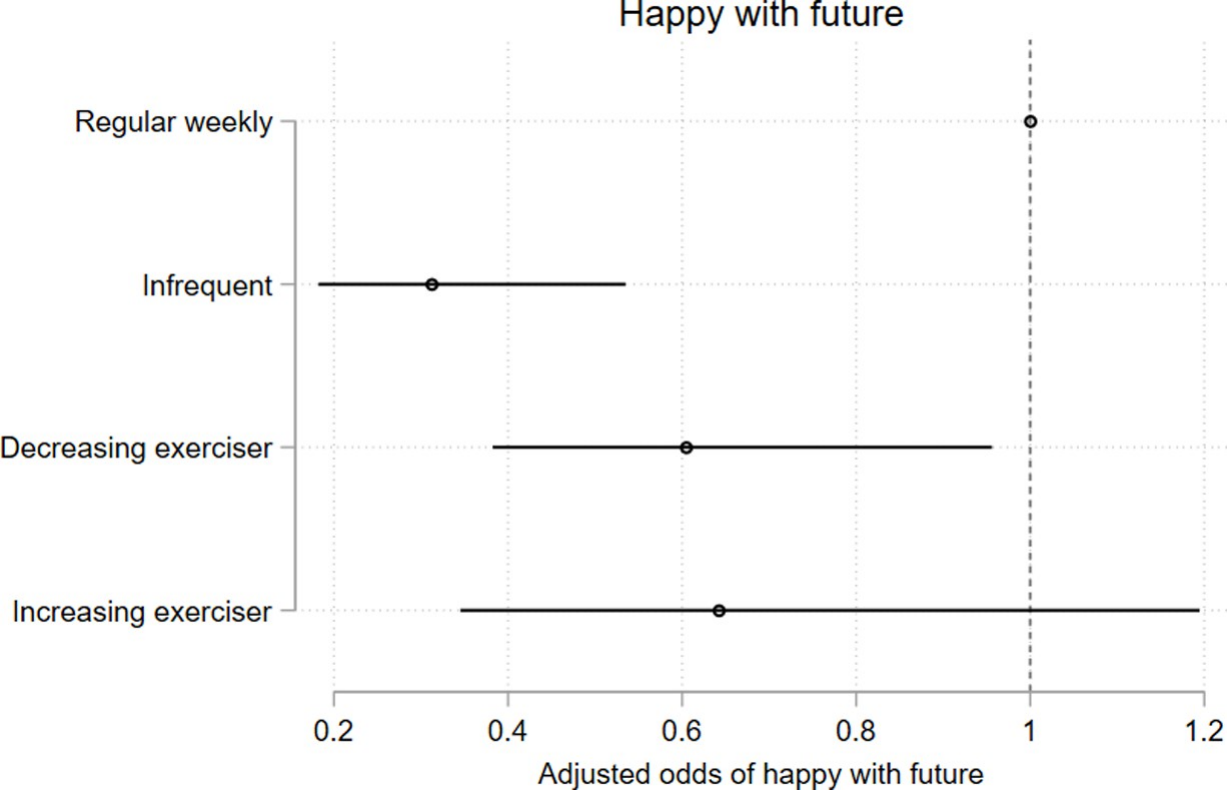
Model 2 trajectory group associations with health-, mental health-, and educational outcomes at age 25. Associations expressed as adjusted odds ratios (aORs) describing the odds of experiencing each outcome of interest for participants who undertake lower levels of recreational physical exercise (infrequent exercisers, decreasing exercisers, increasing exercisers) vs participants undertaking a higher level of recreational physical exercise (*weekly* exercisers). Outcomes are Fig 10) poorer general health; Fig 11) mental illness; Fig 12) overall satisfaction with the future; Fig 13) overall satisfaction with life.

**Fig 13:**
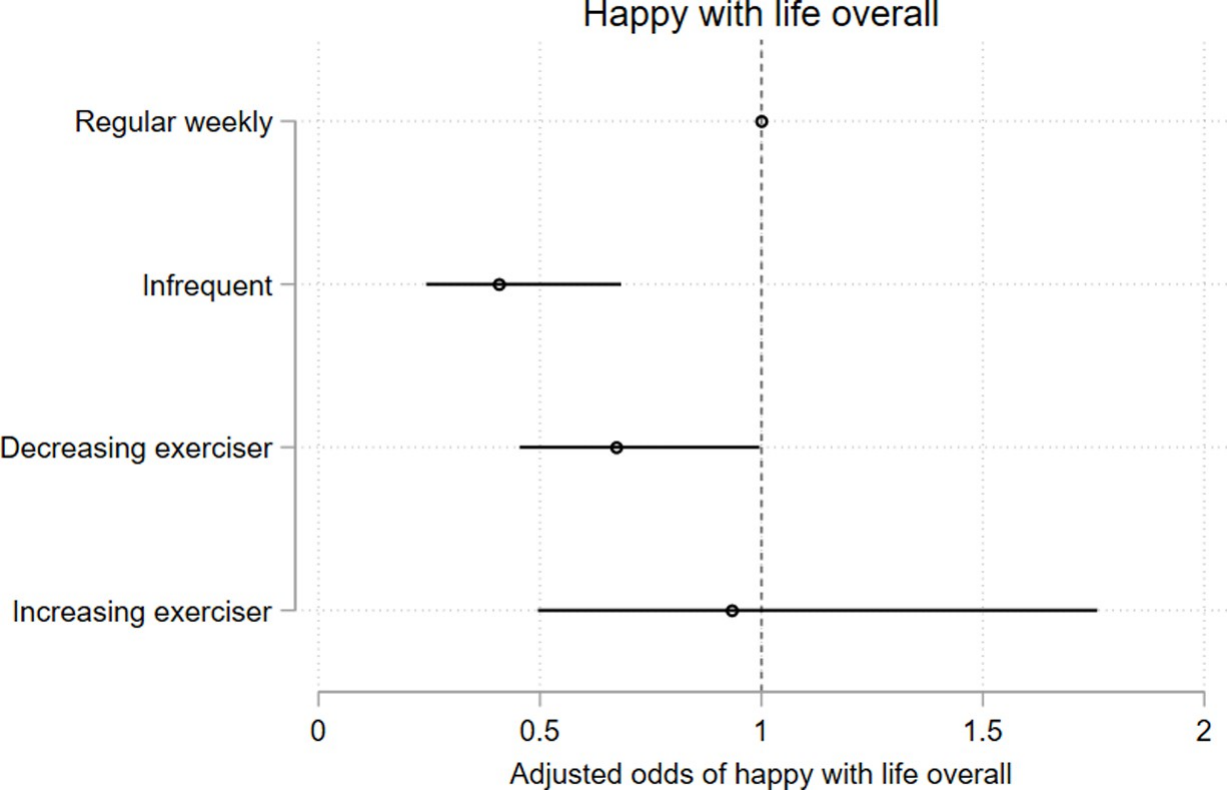
Model 2 trajectory group associations with health-, mental health-, and educational outcomes at age 25. Associations expressed as adjusted odds ratios (aORs) describing the odds of experiencing each outcome of interest for participants who undertake lower levels of recreational physical exercise (infrequent exercisers, decreasing exercisers, increasing exercisers) vs participants undertaking a higher level of recreational physical exercise (*weekly* exercisers). Outcomes are Fig 10) poorer general health; Fig 11) mental illness; Fig 12) overall satisfaction with the future; Fig 13) overall satisfaction with life.

Membership in less-than weekly exercise trajectory groups were negatively associated with measures of general health, psychological distress, and life satisfaction at age 25, and these associations persisted after adjusting for the potentially confounding effects of gender, ethnicity, participants’ ISEI score, and other identified predictor variables (Figs 10–13 and S6 Table). The associations between trajectory-group membership and the attainment of school and post-school qualifications, and with participation in the labour force did not persist following adjustment. (S9–S11 Figs and S6 Table).

Compared to the *weekly* exercise trajectory, *infrequent exercisers*, *declining exercisers*, *and increasing-exercisers* had significantly higher odds of poorer self-reported general health (results reported as adjusted Odds Ratio (aOR), 95% CI; p); for *infrequent exercisers* = 2.57, 95% CI 2.02, 3.28; p < .0001; for *declining exercisers* = 1.97, 95% CI 1.66, 2.34; p < .0001; aOR for *increasing exercisers* = 1.81, 95% CI 1.46, 2.24; p < .0001)(Fig 10) and higher odds of elevated K6 scores suggestive of probable mental illness (aOR for *infrequent exercisers* = 2.42, 95% CI 1.55, 3.78; p < .0001; aOR for *declining exercisers* = 2.19, 95% CI 1.64, 2.93; p < .0001; aOR for *increasing exercisers* = 1.59, 95% CI 1.03, 2.47; p = 0.036)(Fig 11). Young people in the *infrequent exercisers*, *declining exercisers*, and *increasing exercisers* groups were less likely to feel happy about the future; aOR for *infrequent exercisers* = 0.25, 95% CI 0.15, 0.42; p < .0001; aOR for *declining exercisers* = 0.51, 95% CI 0.33, 0.79; p = 0.002; aOR for *increasing exercisers* = 0.54, 95% CI 0.30, 0.97; p = 0.041)(Fig 12), while the *infrequent exercisers* and *declining exercisers* groups were less likely to be satisfied with life overall (aOR for *infrequent exercisers* = 0.33, 95% CI 0.20, 0.54; p < .0001; adjusted OR for *declining exercisers* = 0.57, 95% CI 0.39, 0.84; p = 0.004)(Fig 13).

For health-, mental health-, and life satisfaction measures we observed stepped decreases in the odds of adverse outcomes, with the *infrequent exercisers* group experiencing the highest disadvantage relative to *weekly* exercise group, followed by *declining exercisers* and *increasing exercisers* (Figs 10–13) (there was little variability in the total n available for each variable due to incomplete data (n range 3145–3343)).

With respect to educational attainment at age 25, membership in the *increasing exercisers* group was associated with lower odds of year 12 high school completion, compared with *weekly* exercisers (adjusted OR for *increasing exercisers* = 0.43, 95% CI 0.23, 0.80; p < .008) (S9 Fig). Young people in the *decreasing exerciser* group were less likely than *weekly* exercisers to participate in the workforce at age 25 (adjusted OR for *decreasing exercisers* = 0.58, 95% CI 0.35, 0.95; p < .031) (S11 Fig). There were no other associations of exercise trajectory membership with measures of post school educational or occupational attainment (see S10 Fig).

## 4. Discussion

To our knowledge, this study is the largest longitudinal investigation to date of long-term recreational exercise patterns in young people during the transition from adolescence to early adulthood. Using GBTM, we delineated four distinct patterns of exercise behaviours over the 8-year period from age 16 to 24 in 2 models, exercise guideline adherent exercise (model 1), and non-exercise guideline adherent exercise engagement (model 2). Factors predicting long-term adherence to daily recreational exercise as recommended by WHO guidelines were male gender, higher levels of academic self-efficacy, higher levels of sports participation, less time spent watching TV, identifying as indigenous, and lower parental socioeconomic status (SES). Higher academic literacy at age 15 was associated with a decreased likelihood of adhering to WHO guidelines over the subsequent 8 years, and with increased odds of reducing exercise from daily levels, over time.

Conversely, factors at age 15 predicting irregular (*less than weekly*) exercise over time were female gender, lower levels of academic self-efficacy, lower levels of sports participation, more time spent watching TV, and lower parental socioeconomic status (SES). Higher academic literacy at age 15 increased the risk of being a member of both the *declining exerciser* and the *increasing exerciser* groups.

At age 25, participants in the *guideline adherent* exercise trajectory were more likely to report better general health, compared with those who never met guideline frequency (model 1), while young people with persistent *less than weekly* exercise were more likely to report poorer outcomes in measures of general health, mental health, and life satisfaction compared to young people with more regular (at least once weekly) exercise (model 2). Measures of educational attainment or participation in the labour force were not associated with exercise trajectory membership.

Earlier studies applying GBTM to primary school children to about 18 years of age found evidence latent classes of exercise behaviours with best model fit were of 2 to 4 groups in size [33, 34]. Overall, these studies suggest that physical activity levels decline from childhood to the age of about 16 in most children. Our model 2 indicated that between the ages of 16 and 24, self-reported exercise patterns were relatively stable at a modest level (once weekly or more) for the majority of participants, consistent with other studies in older adolescents reporting that the majority clustered within stable trajectories of low to moderate exercise frequency [35]. The notion that long-term exercise habits are already quite firmly established by late adolescence is further supported by studies that followed young adults into mid-adulthood, showing that most participants were either stably “moderately active” [36], “persistently low active” [37], or “inactive” [38]. Notably, the latter two studies also support our finding of discernible smaller groups who show changes in exercise behaviours in late adolescence and adulthood (i.e., our “increasers” and “decreasers”).

Our finding that female gender is the strongest risk factor for insufficient and less consistent recreational physical activity between the ages of 16 and 24 (in both trajectory models 1 and 2) echoes previous reports that young females are generally less physically active than males [39], and that they are at higher risk of decreasing activity levels over time [40]. Multiple factors have been reported that put young females at disadvantage, including reduced opportunity, lower access, and lack of sports diversity, but also divergent parental and cultural expectations, stereotypes, and role models [41]. Psychological factors such as perceived sports competency may play an additional role [42]. Therefore, the active encouragement and validation of female sports participation across institutions and media are required to counteract the dropout of female adolescents from regular recreational physical activity and exercise.

We identified that higher academic self-efficacy at age 15 predicted greater exercise participation and more consistent long-term exercise behaviours. Self-efficacy is an individual’s belief in their ability to perform a specific action required to attain a desired outcome, and is thought to lead to setting higher goals, investing more effort in the pursuit of these goals, and persisting through barriers or setbacks [43]. In adolescents and young adults, associations between high levels of self-efficacy and higher physical activity levels have been shown [44], and self-efficacy is thought to be one of the driving cognitive factors for engagement in regular exercise [45]. Psychological health interventions focusing specifically on self-efficacy have shown some promise in improving exercise engagement in this age group in the short term [46]. Our findings suggest that such interventions, delivered around the age of 15, may also have a positive impact on the establishment of long-term exercise behaviours.

Time spent exercising and time spent watching television at age 15 were additional predictors for participation in physical exercise. Previous studies have found that sports participation in adolescence predicts physical activity in adulthood [16, 47]. An inverse relationship between longitudinal patterns of television viewing and physical exercise in teenagers has also been reported [48]. Our findings underscore the importance of establishing regular recreational exercise habits in early adolescence, and of limiting screen-based leisure activities to prevent ongoing inactivity or a decline in exercise.

While parental socioeconomic factors have been reported as important for exercise engagement in children and younger adolescents [34], we found they had less impact in our older, more independent cohort of 16–24 year-olds. Our finding of higher academic literacy as a risk factor for lower and less consistent longitudinal exercise groups is interesting, and perhaps reflects competing academic demands (e.g., university entry). Increased exercise opportunities for high academic achievers, for example through programmes in senior high school and university, should be considered.

The favourable outcomes at age 25 for young people engaging in weekly recreational exercisers are in keeping with previous prospective studies in adult populations that have shown benefit from exercise in measures of physical [49] and mental health [10]. In adolescents and young adults, previous longitudinal studies have found advantages for regular or increasing exercisers for educational outcomes [50] and for health-related quality of life measures [15]. Recently, the first longitudinal study reporting objectively measured physical activity in 12–18 year-olds convincingly demonstrated protective effects against depressive symptoms at age 18 [35]. In contrast, one study reported that frequent physical exercise at age 14 was not associated with affective, anxiety, or substance use disorder diagnoses at age 21 [51]. We argue that trajectory analysis of repeatedly-measured data provides a more accurate measure of behaviour patterns over time and is hence more sensitive for predicting longitudinal outcomes. As might be expected, outcome differences between those reporting ongoing daily exercise (“guideline”) and those with less regular regimes were less pronounced. Nevertheless, long-term daily exercisers reported significantly better subjective general health at age 25, an age where physical health concerns are not particularly prevalent. We would expect that these subtle health advantages of daily exercise increase in older age groups.

### 4.1 Strengths and limitations

We used data from a national prospective cohort study with a large sample size and long duration of follow up. As expected, participation rates declined over the course of the study, and non-random dropout may be a source of bias. S5 and S6 Tables provide a comparison of baseline characteristics and trajectory group membership between those with missing outcome data and those with outcome data available. Exercise data were self-reported and are likely less reliable than more objective measures of exercise participation such as wearable actigraphy devices [52]. Objective monitoring of exercise over long periods such as the 8 years covered in our study will become more feasible in the future as wearable technology becomes increasingly widespread. The design of the exercise questionnaire used for LSAY led to skewing of categories towards very low exercise frequencies and prompted us to dichotomize exercise behaviours into binary variables used in two separate longitudinal models. We believe that this approach captures behaviours most relevant from a public health perspective: adherence to international physical activity guidelines reflected in model 1; and very irregular exercise engagement reflected in model 2). However, future iterations of population studies such as LSAY should consider more precise scales to measure frequency of recreational exercise. We are unable to rule out residual confounding by factors not assessed in the survey with respect to the predictors of trajectory-group assignment and/or associations with the selected outcomes. These may include motivational, psychological or physiological resilience factors or life events that predispose individuals to particular patterns of exercise and achievement over the lifespan.

Our study design does not allow for conclusions about a causal relationship between exercise and our reported outcomes. It is possible that young people who enjoy better health, mental health, and educational success are also more prone to regular recreational exercise, or that more regular exercise is associated with other health behaviours that moderate our reported effects, such as smoking [53].

### 4.2 Implications of findings

Our findings could help inform preventive health initiatives in late adolescence and early adulthood and are applicable to high-school-, university-, and vocational college settings. While a higher level of consistent recreational exercise was associated with superior long-term outcomes across the measured domains, our findings also indicate that young people who increase exercise participation over time to even moderate levels (i.e., once weekly or more) experience advantages compared to those who are consistently inactive or whose participation decreases over time. Hence, the commencement and maintenance of regular exercise should be recommended and promoted at all ages. Our findings point to subgroups of young people who might benefit from more tailored exercise-promotion interventions. These include females, those with lower levels of self-efficacy, those who are inactive at age 15 with high screen time, those from lower SES families, and high academic achievers. Future research needs to identify the specific barriers and enablers of exercise participation for these groups.

## 5. Conclusions

We have modelled patterns of recreational exercise participation in late adolescence and early adulthood as distinct latent trajectories to gain policy-relevant insights about exercise participation during the transition from adolescence to adulthood. High school, university, and vocational colleges are important settings to implement interventions that support young people in establishing regular physical activity. Our study identified subgroups of young people that may especially benefit from targeted interventions. We showed that regular long-term regular exercise participation during the transition to adulthood may be associated with measurable benefits for health and mental health in young adults.

## Supporting information

S1 Fig Distribution of observed responses for recreational exercise participation (infrequent vs regular weekly exercise) (model 1) (S1 Fig), and distribution of observed responses for recreational exercise participation (weekly vs less than weekly exercise) (model 2) (S2 Fig). (TIF)

S2 Fig Distribution of observed responses for recreational exercise participation (infrequent vs regular weekly exercise) (model 1) (S1 Fig), and Distribution of observed responses for recreational exercise participation (weekly vs less than weekly exercise) (model 2) (S2 Fig). (TIF)

S3 Fig Model 1 trajectory group associations with health-, mental health-, and educational outcomes at age 25. Associations expressed as adjusted odds ratios (ORs) describing the odds of experiencing each outcome of interest for participants who undertake less than daily recreational physical exercise (the *never guideline*, *guideline dropouts*, and *towards guideline* exercise trajectories) vs participants undertaking the recommended level of recreational physical exercise (*guideline* exercisers). There were no associations between less than guideline levels of recreational exercise and (S3 Fig) mental illness; (S4 Fig) overall satisfaction with life; (S5 Fig) satisfaction with the future; (S6 Fig) completing high school year 12, or higher; (S7 Fig) attaining any post-school qualification; (S8 Fig) participation in the labour force.
(TIF)

S4 Fig Model 1 trajectory group associations with health-, mental health-, and educational outcomes at age 25. Associations expressed as adjusted odds ratios (ORs) describing the odds of experiencing each outcome of interest for participants who undertake less than daily recreational physical exercise (the *never guideline*, *guideline dropouts*, and *towards guideline* exercise trajectories) vs participants undertaking the recommended level of recreational physical exercise (*guideline* exercisers). There were no associations between less than guideline levels of recreational exercise and (S3 Fig) mental illness; (S4 Fig) overall satisfaction with life; (S5 Fig) satisfaction with the future; (S6 Fig) completing high school year 12, or higher; (S7 Fig) attaining any post-school qualification; (S8 Fig) participation in the labour force.
(TIF)

S5 Fig Model 1 trajectory group associations with health-, mental health-, and educational outcomes at age 25. Associations expressed as adjusted odds ratios (ORs) describing the odds of experiencing each outcome of interest for participants who undertake less than daily recreational physical exercise (the *never guideline*, *guideline dropouts*, and *towards guideline* exercise trajectories) vs participants undertaking the recommended level of recreational physical exercise (*guideline* exercisers). There were no associations between less than guideline levels of recreational exercise and (S3 Fig) mental illness; (S4 Fig) overall satisfaction with life; (S5 Fig) satisfaction with the future; (S6 Fig) completing high school year 12, or higher; (S7 Fig) attaining any post-school qualification; (S8 Fig) participation in the labour force.
(TIF)

S6 Fig Model 1 trajectory group associations with health-, mental health-, and educational outcomes at age 25. Associations expressed as adjusted odds ratios (ORs) describing the odds of experiencing each outcome of interest for participants who undertake less than daily recreational physical exercise (the *never guideline*, *guideline dropouts*, and *towards guideline* exercise trajectories) vs participants undertaking the recommended level of recreational physical exercise (*guideline* exercisers). There were no associations between less than guideline levels of recreational exercise and (S3 Fig) mental illness; (S4 Fig) overall satisfaction with life; (S5 Fig) satisfaction with the future; (S6 Fig) completing high school year 12, or higher; (S7 Fig) attaining any post-school qualification; (S8 Fig) participation in the labour force.
(TIF)

S7 Fig Model 1 trajectory group associations with health-, mental health-, and educational outcomes at age 25. Associations expressed as adjusted odds ratios (ORs) describing the odds of experiencing each outcome of interest for participants who undertake less than daily recreational physical exercise (the *never guideline*, *guideline dropouts*, and *towards guideline* exercise trajectories) vs participants undertaking the recommended level of recreational physical exercise (*guideline* exercisers). There were no associations between less than guideline levels of recreational exercise and (S3 Fig) mental illness; (S4 Fig) overall satisfaction with life; (S5 Fig) satisfaction with the future; (S6 Fig) completing high school year 12, or higher; (S7 Fig) attaining any post-school qualification; (S8 Fig) participation in the labour force.
(TIF)

S8 Fig Model 1 trajectory group associations with health-, mental health-, and educational outcomes at age 25. Associations expressed as adjusted odds ratios (ORs) describing the odds of experiencing each outcome of interest for participants who undertake less than daily recreational physical exercise (the *never guideline*, *guideline dropouts*, and *towards guideline* exercise trajectories) vs participants undertaking the recommended level of recreational physical exercise (*guideline* exercisers). There were no associations between less than guideline levels of recreational exercise and (S3 Fig) mental illness; (S4 Fig) overall satisfaction with life; (S5 Fig) satisfaction with the future; (S6 Fig) completing high school year 12, or higher; (S7 Fig) attaining any post-school qualification; (S8 Fig) participation in the labour force.
(TIF)

S9 Fig Model 2 trajectory group associations with health-, mental health-, and educational outcomes at age 25. Associations expressed as adjusted odds ratios (aORs) describing the odds of experiencing each outcome of interest for participants who undertake lower levels of recreational physical exercise (infrequent exercisers, decreasing exercisers, increasing exercisers) vs participants undertaking a higher level of recreational physical exercise (*weekly* exercisers). There were no associations of exercise trajectory membership with S9 Fig) completion of year 12 or higher; S10 Fig) the attainment of a post school qualification; or Fig 15) labour force participation at age 25.
(TIF)

S10 Fig Model 2 trajectory group associations with health-, mental health-, and educational outcomes at age 25. Associations expressed as adjusted odds ratios (aORs) describing the odds of experiencing each outcome of interest for participants who undertake lower levels of recreational physical exercise (infrequent exercisers, decreasing exercisers, increasing exercisers) vs participants undertaking a higher level of recreational physical exercise (*weekly* exercisers). There were no associations of exercise trajectory membership with S9 Fig) completion of year 12 or higher; S10 Fig) the attainment of a post school qualification; or Fig 15) labour force participation at age 25.
(TIF)

S11 Fig Model 2 trajectory group associations with health-, mental health-, and educational outcomes at age 25. Associations expressed as adjusted odds ratios (aORs) describing the odds of experiencing each outcome of interest for participants who undertake lower levels of recreational physical exercise (infrequent exercisers, decreasing exercisers, increasing exercisers) vs participants undertaking a higher level of recreational physical exercise (*weekly* exercisers). There were no associations of exercise trajectory membership with S9 Fig) completion of year 12 or higher; S10 Fig) the attainment of a post school qualification; or Fig 15) labour force participation at age 25.
(TIF)

S1 Table Summary statistics for continuous predictors for model 1 trajectory groups. (DOCX)

S2 Table Summary statistics for binary and categorical predictors for model 1 trajectory groups. (DOCX)

S3 Table Summary statistics for continuous variables for model 2 trajectory groups. (DOCX)

S4 Table Summary statistics for binary and categorical predictors for model 2 trajectory groups. Percentages may not equal 100 due to rounding.
(DOCX)

S5 Table Summary statistics of daily vs less than daily (model 1) trajectory group associations with outcomes at age 25. (DOCX)

S6 Table Summary statistics of weekly vs less than weekly (model 2) trajectory group associations with outcomes at age 25. (DOCX)
