# Supplementary figures and images for: Long-term recreational exercise patterns in adolescents and young adults: Trajectory predictors and associations with health, mental-health, and educational outcomes
Source: PLoS One. 2024 Mar 21;19(3):e0284660. doi: 10.1371/journal.pone.0284660 (PMC10956783; doi:10.1371/journal.pone.0284660)

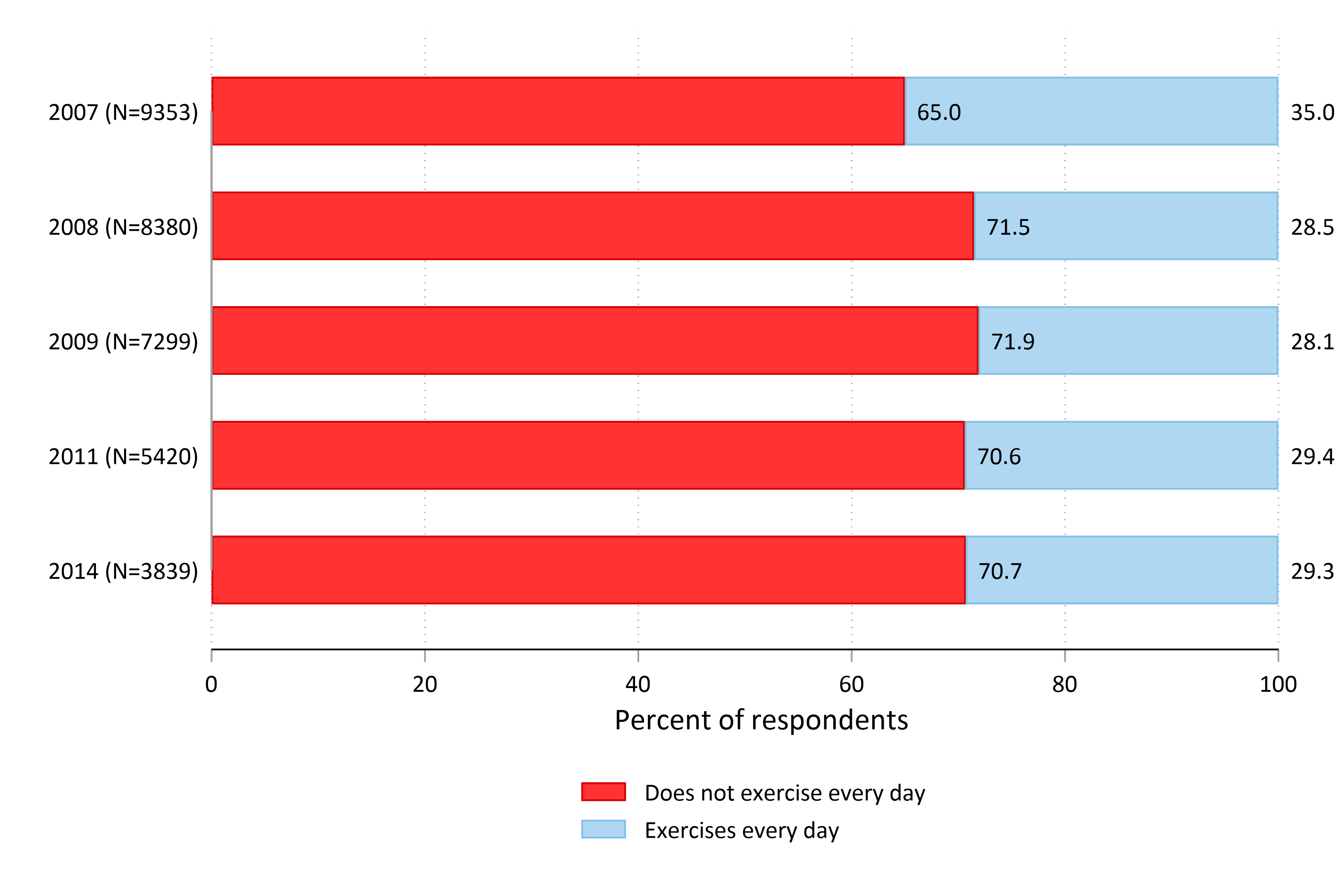

Supplement: S1 Fig — (TIF) [file pone.0284660.s001.tif]

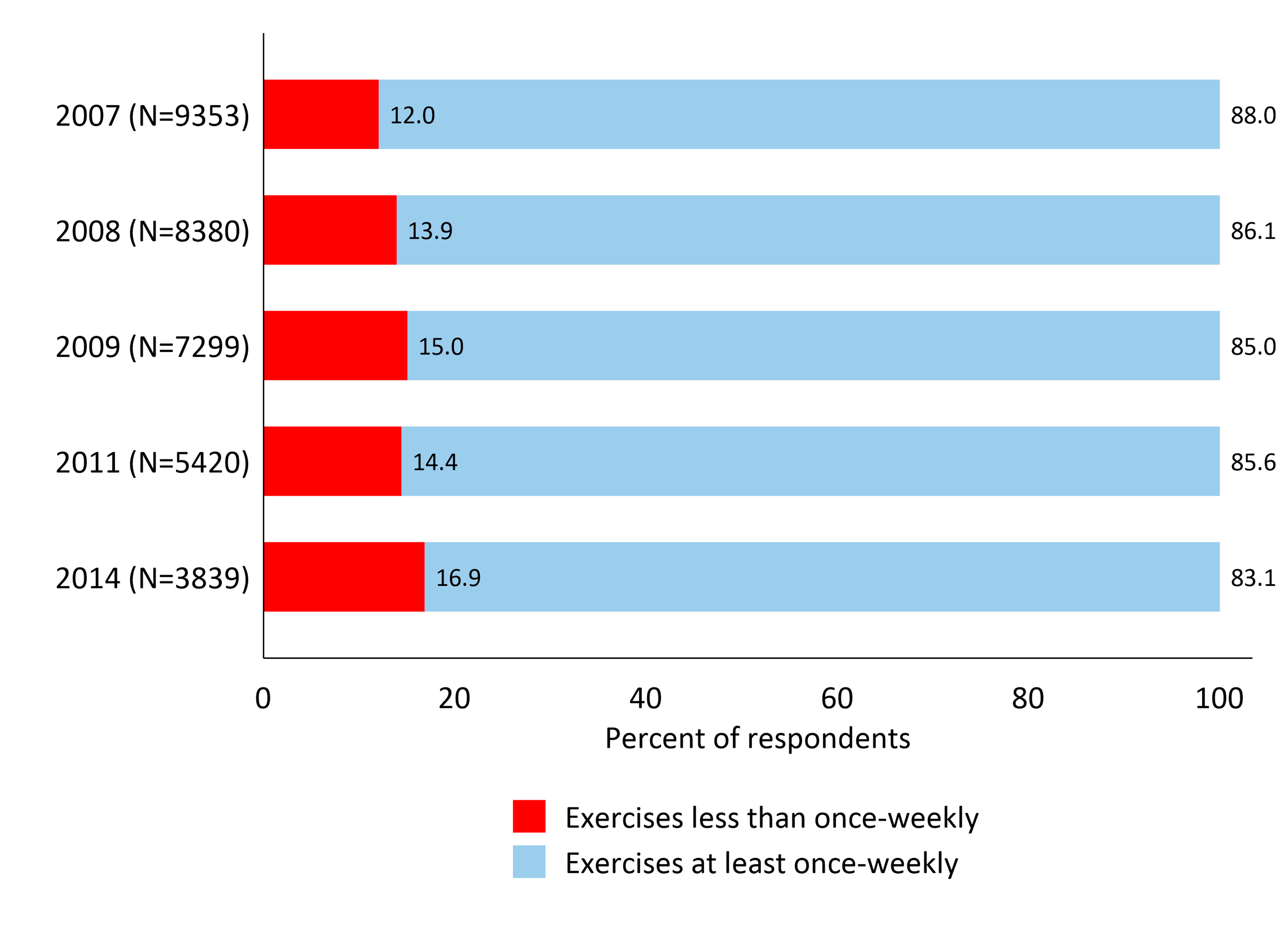

Supplement: S2 Fig — (TIF) [file pone.0284660.s002.tif]

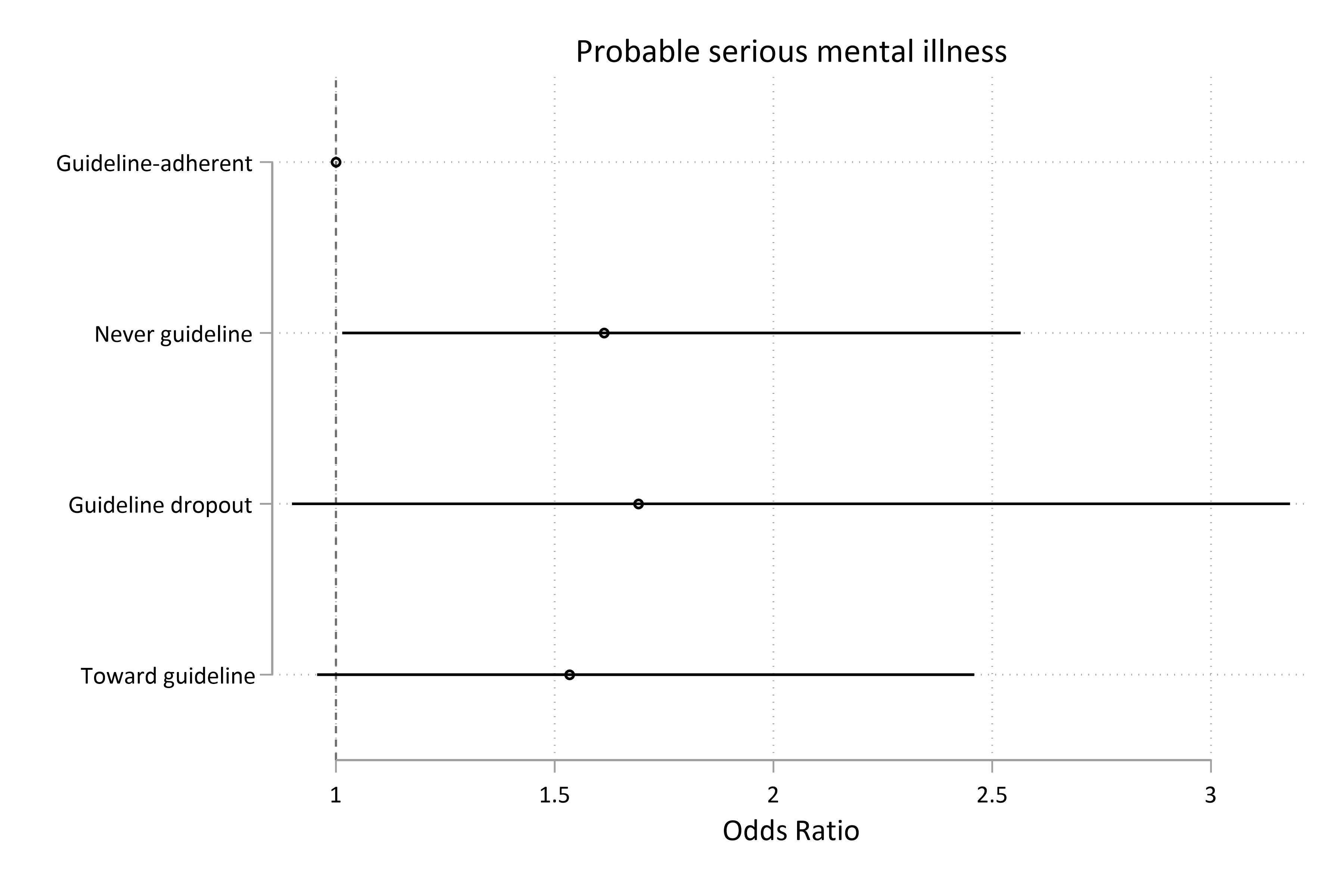

Supplement: S3 Fig — Associations expressed as adjusted odds ratios (ORs) describing the odds of experiencing each outcome of interest for participants who undertake less than daily recreational physical exercise (the never guideline, guideline dropouts, and towards guideline exercise trajectories) vs participants undertaking the recommended level of recreational physical exercise (guideline exercisers). There were no associations between less than guideline levels of recreational exercise and (S3 Fig) mental illness; (S4 Fig) overall satisfaction with life; (S5 Fig) satisfaction with the future; (S6 Fig) completing high school year 12, or higher; (S7 Fig) attaining any post-school qualification; (S8 Fig) participation in the labour force. (TIF) [file pone.0284660.s003.tif]

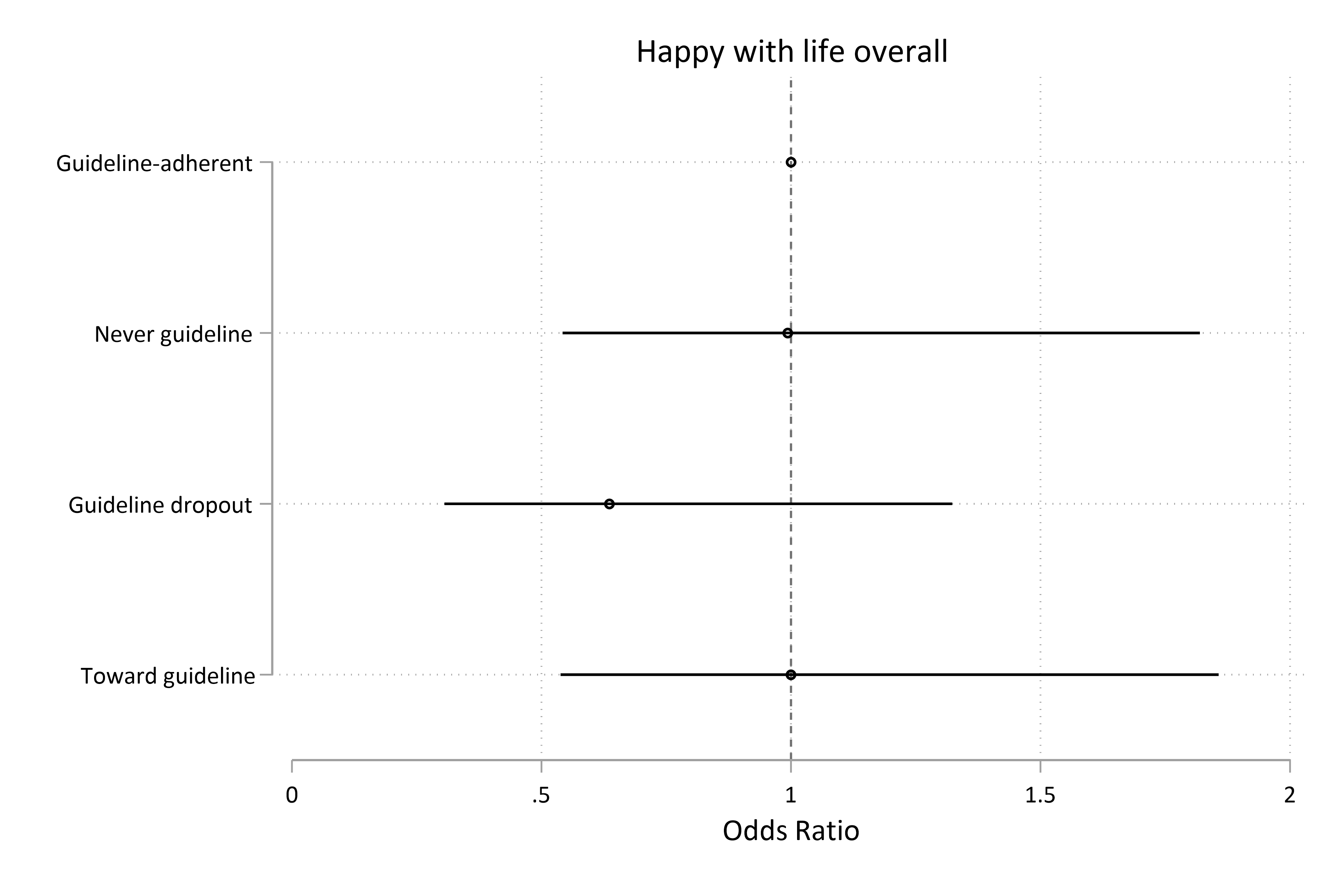

Supplement: S4 Fig — Associations expressed as adjusted odds ratios (ORs) describing the odds of experiencing each outcome of interest for participants who undertake less than daily recreational physical exercise (the never guideline, guideline dropouts, and towards guideline exercise trajectories) vs participants undertaking the recommended level of recreational physical exercise (guideline exercisers). There were no associations between less than guideline levels of recreational exercise and (S3 Fig) mental illness; (S4 Fig) overall satisfaction with life; (S5 Fig) satisfaction with the future; (S6 Fig) completing high school year 12, or higher; (S7 Fig) attaining any post-school qualification; (S8 Fig) participation in the labour force. (TIF) [file pone.0284660.s004.tif]

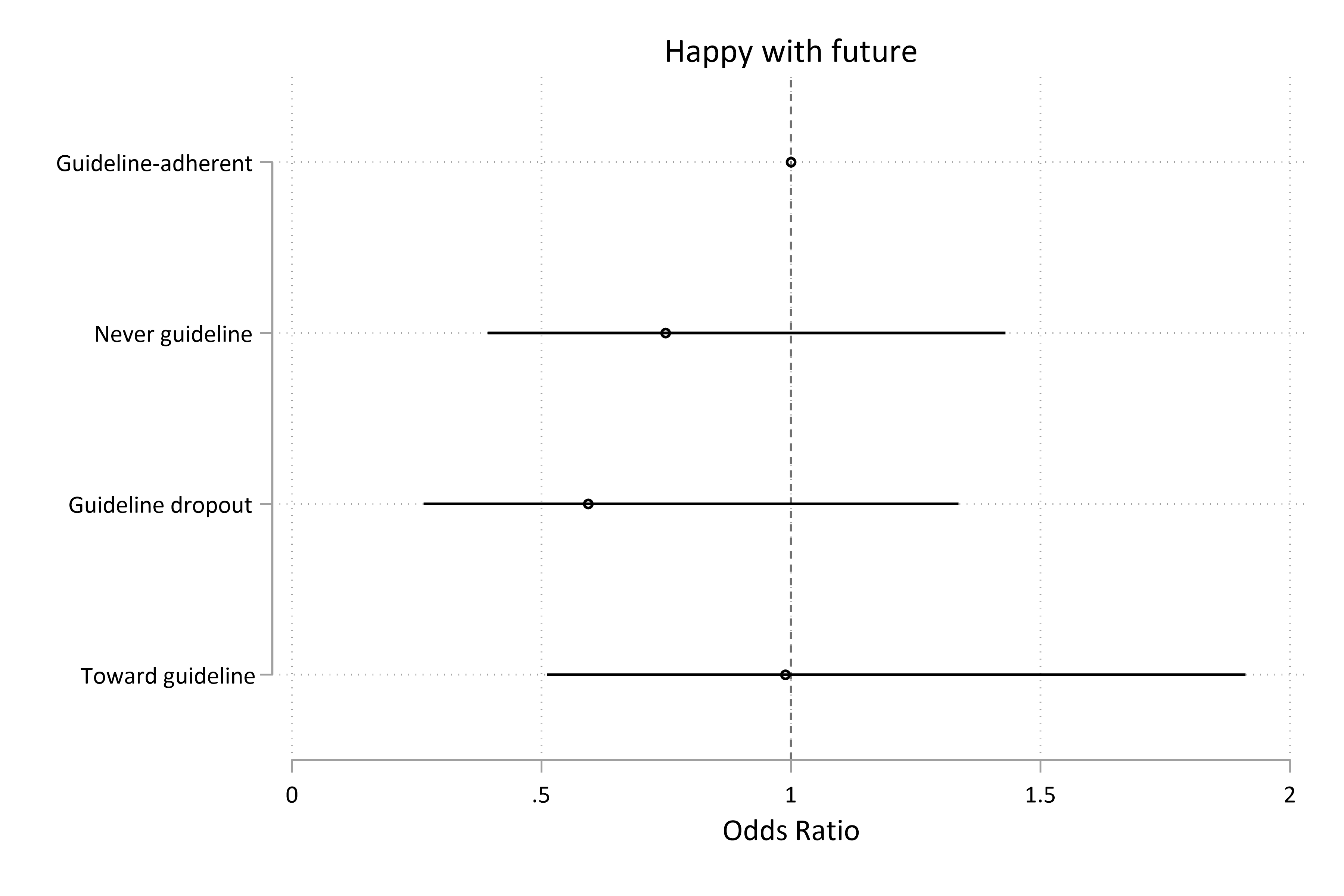

Supplement: S5 Fig — Associations expressed as adjusted odds ratios (ORs) describing the odds of experiencing each outcome of interest for participants who undertake less than daily recreational physical exercise (the never guideline, guideline dropouts, and towards guideline exercise trajectories) vs participants undertaking the recommended level of recreational physical exercise (guideline exercisers). There were no associations between less than guideline levels of recreational exercise and (S3 Fig) mental illness; (S4 Fig) overall satisfaction with life; (S5 Fig) satisfaction with the future; (S6 Fig) completing high school year 12, or higher; (S7 Fig) attaining any post-school qualification; (S8 Fig) participation in the labour force. (TIF) [file pone.0284660.s005.tif]

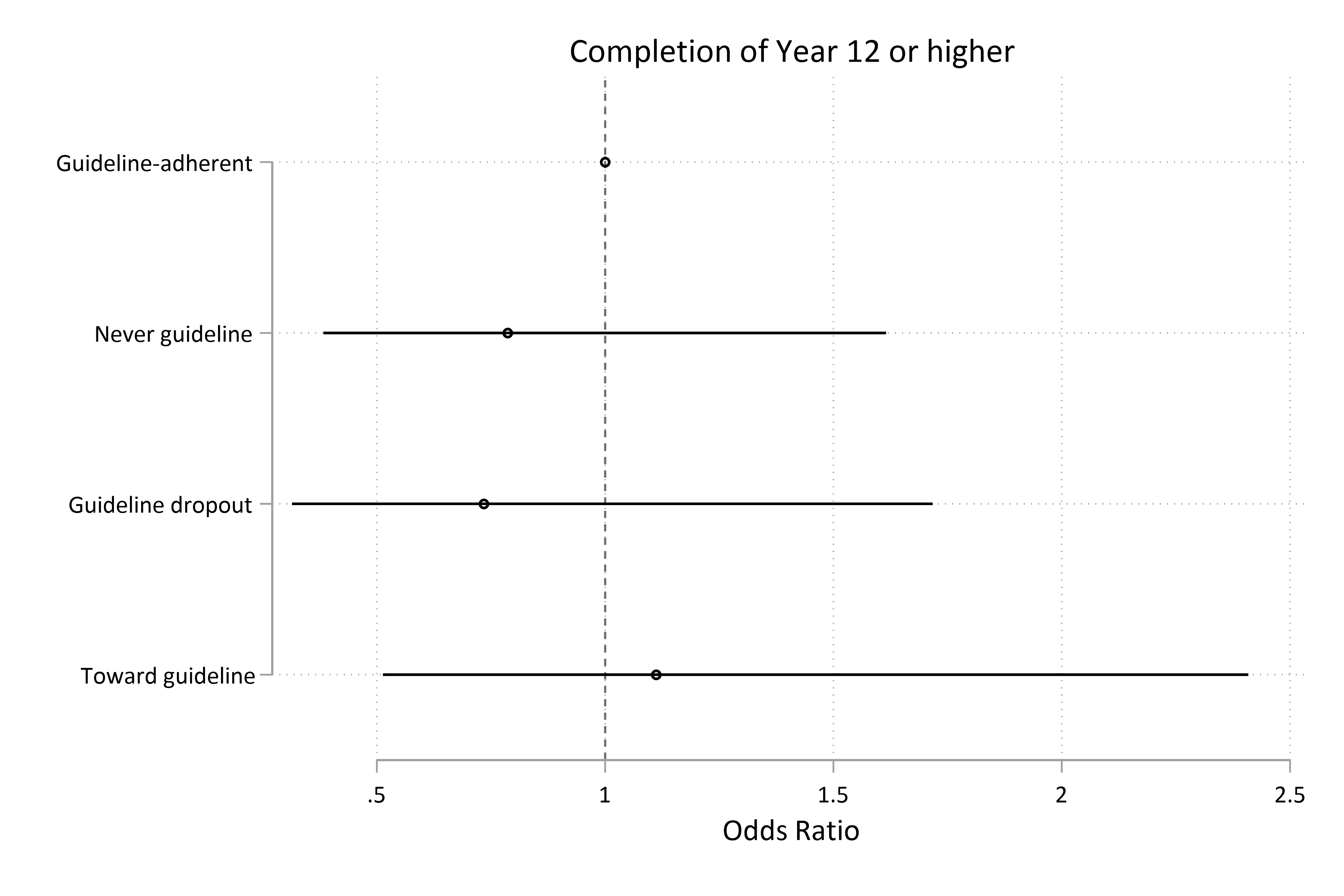

Supplement: S6 Fig — Associations expressed as adjusted odds ratios (ORs) describing the odds of experiencing each outcome of interest for participants who undertake less than daily recreational physical exercise (the never guideline, guideline dropouts, and towards guideline exercise trajectories) vs participants undertaking the recommended level of recreational physical exercise (guideline exercisers). There were no associations between less than guideline levels of recreational exercise and (S3 Fig) mental illness; (S4 Fig) overall satisfaction with life; (S5 Fig) satisfaction with the future; (S6 Fig) completing high school year 12, or higher; (S7 Fig) attaining any post-school qualification; (S8 Fig) participation in the labour force. (TIF) [file pone.0284660.s006.tif]

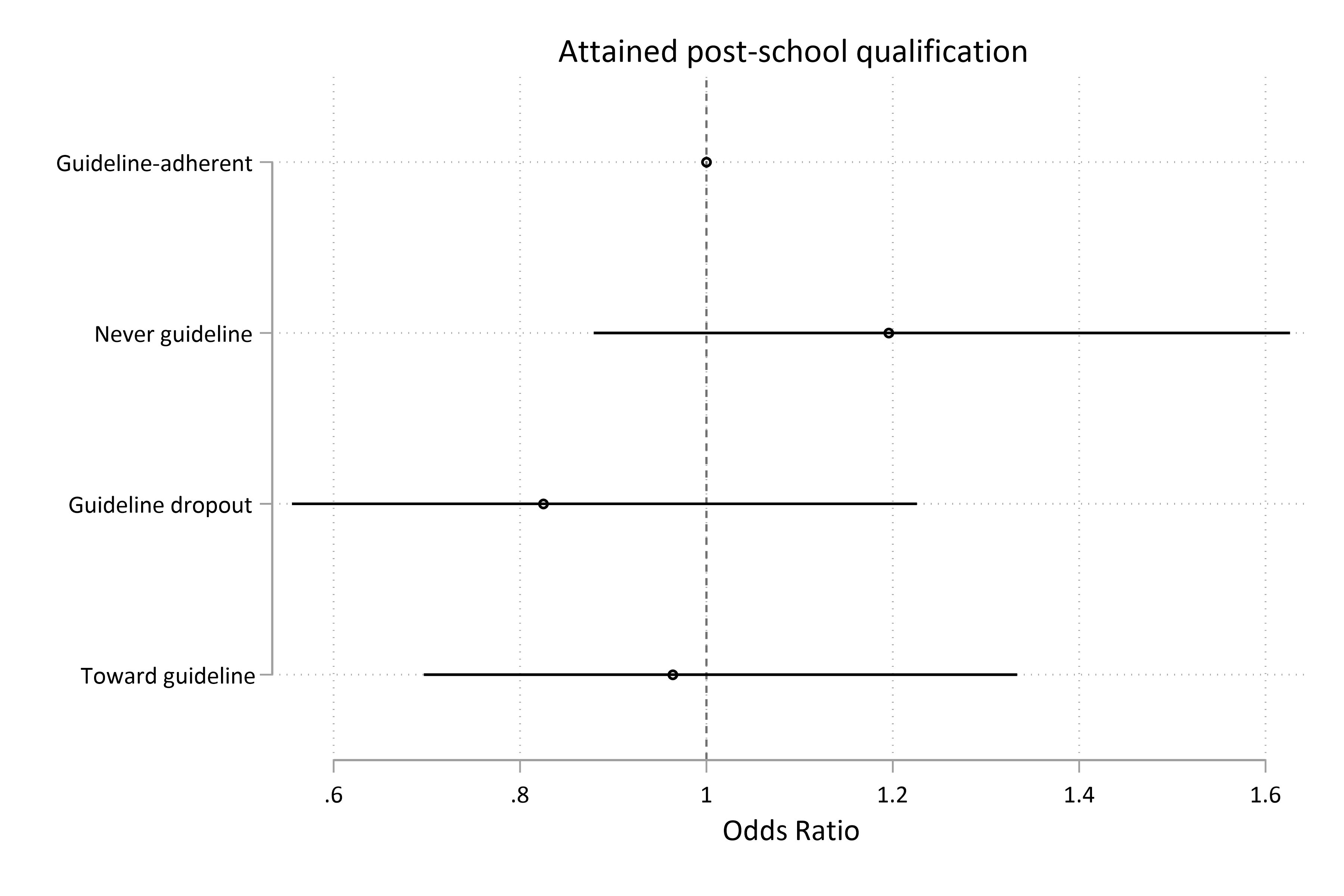

Supplement: S7 Fig — Associations expressed as adjusted odds ratios (ORs) describing the odds of experiencing each outcome of interest for participants who undertake less than daily recreational physical exercise (the never guideline, guideline dropouts, and towards guideline exercise trajectories) vs participants undertaking the recommended level of recreational physical exercise (guideline exercisers). There were no associations between less than guideline levels of recreational exercise and (S3 Fig) mental illness; (S4 Fig) overall satisfaction with life; (S5 Fig) satisfaction with the future; (S6 Fig) completing high school year 12, or higher; (S7 Fig) attaining any post-school qualification; (S8 Fig) participation in the labour force. (TIF) [file pone.0284660.s007.tif]

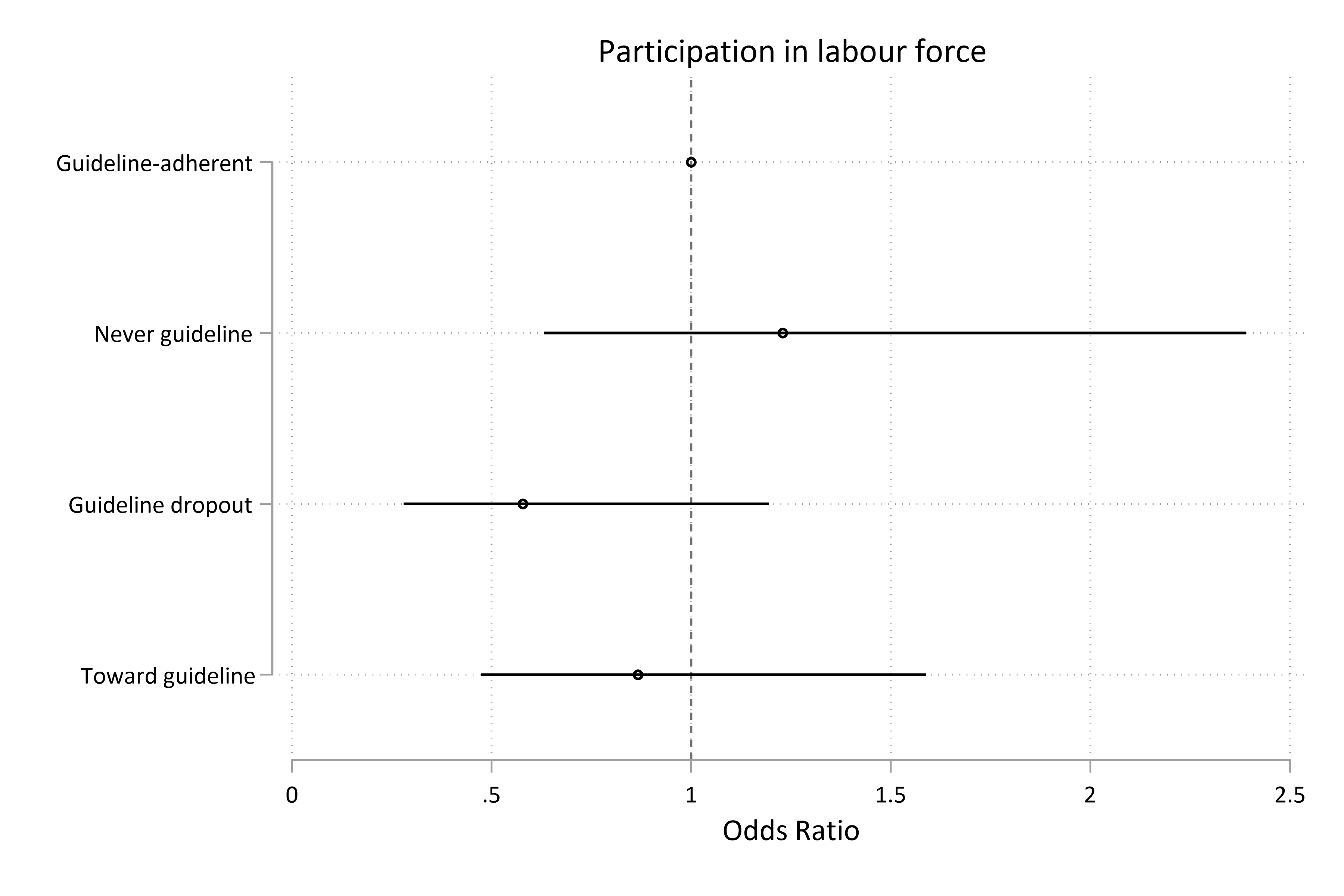

Supplement: S8 Fig — Associations expressed as adjusted odds ratios (ORs) describing the odds of experiencing each outcome of interest for participants who undertake less than daily recreational physical exercise (the never guideline, guideline dropouts, and towards guideline exercise trajectories) vs participants undertaking the recommended level of recreational physical exercise (guideline exercisers). There were no associations between less than guideline levels of recreational exercise and (S3 Fig) mental illness; (S4 Fig) overall satisfaction with life; (S5 Fig) satisfaction with the future; (S6 Fig) completing high school year 12, or higher; (S7 Fig) attaining any post-school qualification; (S8 Fig) participation in the labour force. (TIF) [file pone.0284660.s008.tif]

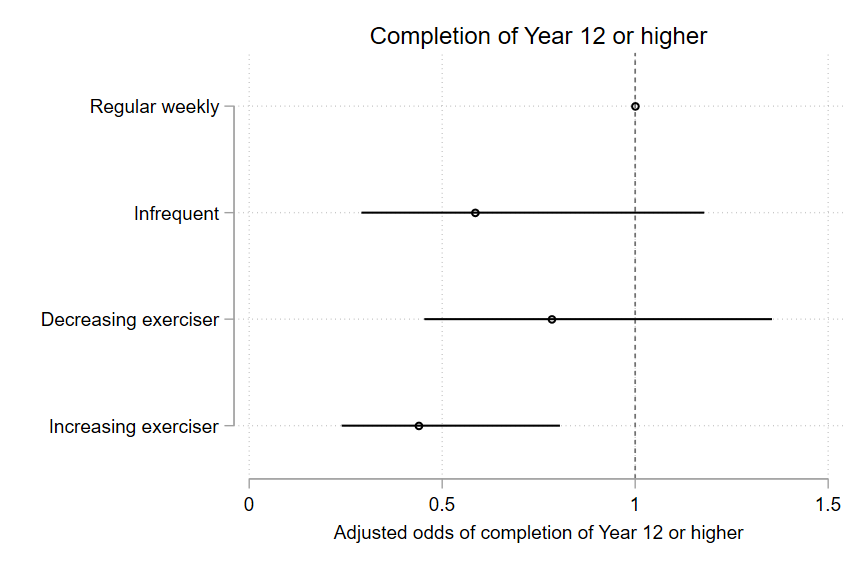

Supplement: S9 Fig — Associations expressed as adjusted odds ratios (aORs) describing the odds of experiencing each outcome of interest for participants who undertake lower levels of recreational physical exercise (infrequent exercisers, decreasing exercisers, increasing exercisers) vs participants undertaking a higher level of recreational physical exercise (weekly exercisers). There were no associations of exercise trajectory membership with S9 Fig) completion of year 12 or higher; S10 Fig) the attainment of a post school qualification; or Fig 15) labour force participation at age 25. (TIF) [file pone.0284660.s009.tif]

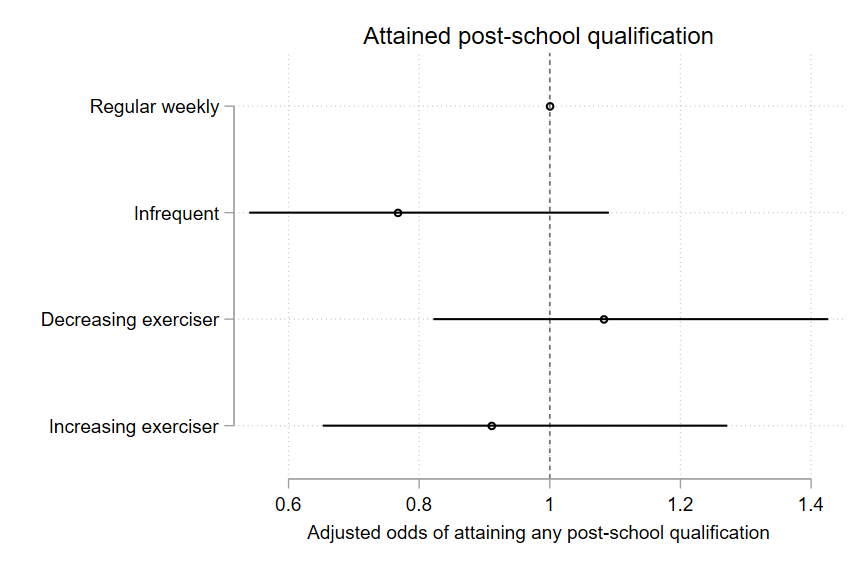

Supplement: S10 Fig — Associations expressed as adjusted odds ratios (aORs) describing the odds of experiencing each outcome of interest for participants who undertake lower levels of recreational physical exercise (infrequent exercisers, decreasing exercisers, increasing exercisers) vs participants undertaking a higher level of recreational physical exercise (weekly exercisers). There were no associations of exercise trajectory membership with S9 Fig) completion of year 12 or higher; S10 Fig) the attainment of a post school qualification; or Fig 15) labour force participation at age 25. (TIF) [file pone.0284660.s010.tif]

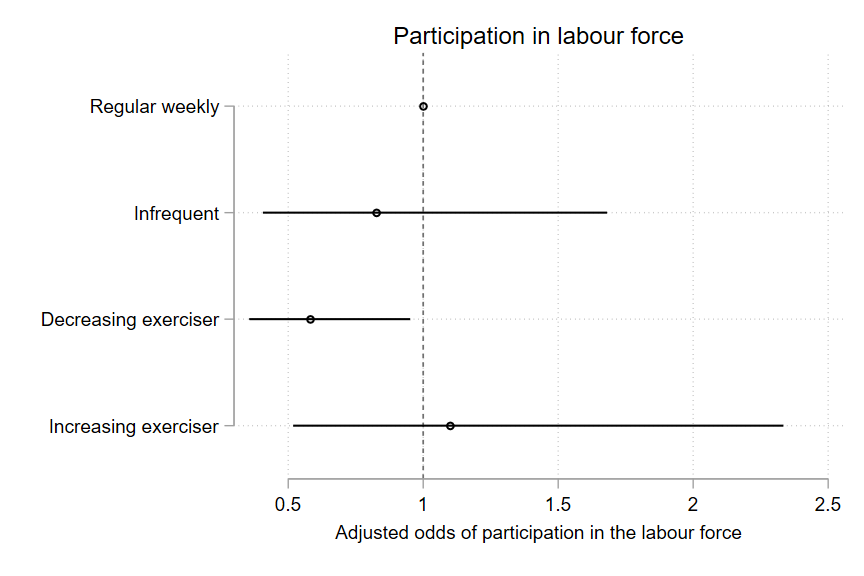

Supplement: S11 Fig — Associations expressed as adjusted odds ratios (aORs) describing the odds of experiencing each outcome of interest for participants who undertake lower levels of recreational physical exercise (infrequent exercisers, decreasing exercisers, increasing exercisers) vs participants undertaking a higher level of recreational physical exercise (weekly exercisers). There were no associations of exercise trajectory membership with S9 Fig) completion of year 12 or higher; S10 Fig) the attainment of a post school qualification; or Fig 15) labour force participation at age 25. (TIF) [file pone.0284660.s011.tif]
