# Supplementary material for: Long-term recreational exercise patterns in adolescents and young adults: Trajectory predictors and associations with health, mental-health, and educational outcomes
Source: PLoS One. 2024 Mar 21;19(3):e0284660. doi: 10.1371/journal.pone.0284660 (PMC10956783; doi:10.1371/journal.pone.0284660)
Supplement: S1 Table — (DOCX) [file pone.0284660.s012.docx]

# Supplementary table 1. Summary statistics for continuous predictors for model 1 trajectory groups.

| Characteristic | Assigned Trajectory Group | | | |
| --- | --- | --- | --- | --- |
|  | Guideline-adherent  (n=1948) | Never guideline  (n=3176) | Guideline drop-out  (n=612) | Towards guideline  (n=3617) |
| International Socio-Economic Index of Occupational Status (ISEI) |  |  |  |  |
| Father’s ISEI score (mean ± SD) | 51.7 ± 22.0 | 50.8 ± 21.5 | 52.2 ± 21.1 | 50.9 ± 22.3 |
| Mother’s ISEI score (mean ± SD) | 53.5 ± 21.6 | 53.0 ± 21.8 | 54.4 ± 21.4 | 53.7 ± 22.5 |
| Time spent each week playing sport (hours) at baseline (mean ± SD) | 9.0 ± 6.9 | 4.5 ± 4.2 | 8.0 ± 6.1 | 5.6 ± 5.3 |
| Time spent each week watching TV (hours) at baseline (mean ± SD) | 10.3 ± 8.9 | 10.8 ± 9.2 | 10.5 ± 9.0 | 10.9 ± 9.2 |
| Academic literacy at baseline (mean ± SD) |  |  |  |  |
| Plausible value in maths, science and reading combined | 7870 ± 1243 | 8199 ± 1220 | 8156 ± 1200 | 7841 ± 1250 |
