# Supplementary material for: Long-term recreational exercise patterns in adolescents and young adults: Trajectory predictors and associations with health, mental-health, and educational outcomes
Source: PLoS One. 2024 Mar 21;19(3):e0284660. doi: 10.1371/journal.pone.0284660 (PMC10956783; doi:10.1371/journal.pone.0284660)
Supplement: S2 Table — (DOCX) [file pone.0284660.s013.docx]

# Supplementary table 2. Summary statistics for binary and categorical predictors for model 1 trajectory groups.

| Characteristic | Assigned Trajectory Group^^^ | | | | | | | |
| --- | --- | --- | --- | --- | --- | --- | --- | --- |
|  | Guideline-adherent  (n=1948) | | Never guideline  (n=3176) | | Guideline drop-out  (n=612) | | Towards guideline  (n=3617) | |
|  | Freq | (%) | Freq | (%) | Freq | (%) | Freq | (%) |
| Gender |  |  |  |  |  |  |  |  |
| Male | 1187 | (60.9) | 1289 | (40.6) | 369 | (60.3) | 1616 | (44.7) |
| Female | 761 | (39.1) | 1887 | (59.4) | 243 | (39.7) | 2001 | (55.3) |
| Indigenous Status |  |  |  |  |  |  |  |  |
| Non-indigenous | 1824 | (93.6) | 3036 | (95.6) | 583 | (95.3) | 3392 | (93.8) |
| Indigenous | 124 | (6.4) | 140 | (4.4) | 29 | (4.7) | 225 | (6.2) |
| Self-efficacy |  |  |  |  |  |  |  |  |
| *“Compared with most students in your year level, how well are you doing in your subjects overall?”* |  |  |  |  |  |  |  |  |
| Below average | 71 | (3.6) | 130 | (4.1) | 17 | (2.8) | 183 | (5.1) |
| About average | 747 | (38.4) | 1300 | (40.9) | 209 | (34.2) | 1639 | (45.3) |
| Above average | 1113 | (57.1) | 1721 | (54.2) | 384 | (62.8) | 1767 | (48.9) |
| Missing/unknown | 17 | (0.9) | 25 | (0.8) | 2 | (0.33) | 28 | (0.77) |
| Self-worth |  |  |  |  |  |  |  |  |
| *“My school is a place where I know I can do well enough to be successful”* |  |  |  |  |  |  |  |  |
| Agree | 1834 | (94.2) | 2972 | (93.6) | 578 | (94.4) | 3324 | (91.9) |
| Disagree | 97 | (5.0) | 184 | (5.8) | 34 | (5.6) | 264 | (7.3) |
| Missing/unknown | 17 | (0.9) | 20 | (0.6) | 0 | (0) | 29 | (0.8) |
| *“I am a success as a student”* |  |  |  |  |  |  |  |  |
| Agree | 1684 | (86.5) | 2692 | 84.8) | 528 | (86.3) | 2985 | (82.5) |
| Disagree | 236 | (12.1) | 449 | (14.1) | 77 | (12.6) | 582 | (16.1) |
| Missing/unknown | 28 | (1.4) | 35 | (1.1) | 7 | (1.1) | 50 | (1.4) |
| *“My school is a place where teachers give me marks I deserve”* |  |  |  |  |  |  |  |  |
| Agree | 1592 | (81.7) | 2714 | (85.5) | 515 | (84.2) | 2941 | (81.3) |
| Disagree | 333 | (17.1) | 432 | (13.6) | 95 | (15.5) | 640 | (17.7) |
| Missing/unknown | 23 | (1.2) | 30 | (0.9) | 2 | (0.3) | 36 | (1.0) |
| Enjoyment of school |  |  |  |  |  |  |  |  |
| *“My school is a place where I get enjoyment from being here”* |  |  |  |  |  |  |  |  |
| Agree | 1471 | (75.5) | 2341 | (73.7) | 459 | (75.0) | 2549 | (70.5) |
| Disagree | 459 | (23.6) | 811 | (25.5) | 150 | (24.5) | 1027 | (28.4) |
| Missing/unknown | 18 | (0.9) | 24 | (0.8) | 3 | (0.5) | 41 | (1.1) |
| *“At school I am given the chance to do interesting work”* |  |  |  |  |  |  |  |  |
| Agree | 1453 | (74.6) | 2414 | (76.0) | 478 | (78.1) | 2607 | (72.1) |
| Disagree | 479 | (24.6) | 743 | (23.4) | 131 | (21.4) | 979 | (27.1) |
| Missing/unknown | 16 | (0.8) | 19 | (0.6) | 3 | (0.49) | 31 | (0.9) |
