# Supplementary material for: Long-term recreational exercise patterns in adolescents and young adults: Trajectory predictors and associations with health, mental-health, and educational outcomes
Source: PLoS One. 2024 Mar 21;19(3):e0284660. doi: 10.1371/journal.pone.0284660 (PMC10956783; doi:10.1371/journal.pone.0284660)
Supplement: S3 Table — (DOCX) [file pone.0284660.s014.docx]

# Supplementary table 3. Summary statistics for continuous variables for model 2 trajectory groups.

| Characteristic | Assigned Trajectory Group | | | |
| --- | --- | --- | --- | --- |
|  | Weekly exerciser  (n=6501) | Infrequent exerciser  (n=778) | Decreasing exerciser  (n=1626) | Increasing exerciser  (n=448) |
| International Socio-Economic Index of Occupational Status (ISEI) |  |  |  |  |
| Father’s ISEI score (mean ± SD) | 47.9 ± 18.1 | 43.8 ± 16.8 | 46.5 ± 17.8 | 47.3 ± 17.7 |
| Mother’s ISEI score (mean ± SD) | 48.4 ± 16.0 | 44.6 ± 15.9 | 46.9 ± 16.8 | 49.3 ± 16.5 |
| Time spent each week playing sport (hours) at baseline (mean ± SD) | 7.0 ± 5.9 | 2.4 ± 3.3 | 4.8 ± 4.9 | 3.5 ± 3.9 |
| Time spent each week watching TV (hours) at baseline (mean ± SD) | 10.6 ± 8.9 | 11.7 ± 10.3 | 10.8 ± 9.2 | 11.0 ± 10.0 |
| Academic literacy at baseline (mean ± SD) |  |  |  |  |
| Plausible value in maths, science and reading combined | 7977 ± 1237 | 7819 ± 1288 | 8036 ± 1276 | 8306 ± 1133 |
