# Supplementary material for: Long-term recreational exercise patterns in adolescents and young adults: Trajectory predictors and associations with health, mental-health, and educational outcomes
Source: PLoS One. 2024 Mar 21;19(3):e0284660. doi: 10.1371/journal.pone.0284660 (PMC10956783; doi:10.1371/journal.pone.0284660)
Supplement: S4 Table — Percentages may not equal 100 due to rounding. (DOCX) [file pone.0284660.s015.docx]

# Supplementary table 4. Summary statistics for binary and categorical predictors for model 2 trajectory groups

Percentages may not equal 100 due to rounding.

| Characteristic | Assigned Trajectory Group^^^ | | | | | | | |
| --- | --- | --- | --- | --- | --- | --- | --- | --- |
|  | Weekly exerciser  (*n*=6501) | | Infrequent exerciser  (*n*=778) | | Decreasing exerciser (*n*=1626) | | Increasing exerciser (*n*=448) | |
|  | Freq | (%) | Freq | (%) | Freq | (%) | Freq | (%) |
| Gender |  |  |  |  |  |  |  |  |
| Male | 3372 | (51.9) | 244 | (31.4) | 686 | (42.2) | 159 | (35.5) |
| Female | 3129 | (48.1) | 534 | (68.6) | 940 | (57.8) | 289 | (64.5) |
| Indigenous Status |  |  |  |  |  |  |  |  |
| Non-indigenous | 6141 | (94.5) | 723 | (92.9) | 1540 | (94.7) | 431 | (96.2) |
| Indigenous | 360 | (5.5) | 55 | (7.1) | 86 | (5.3) | 17 | (3.8) |
| Self-efficacy |  |  |  |  |  |  |  |  |
| *“Compared with most students in your year level, how well are you doing in your subjects overall?”* |  |  |  |  |  |  |  |  |
| Below average | 241 | (3.7) | 57 | (7.3) | 79 | (4.9) | 24 | (5.4) |
| About average | 2605 | (40.1) | 390 | (50.1) | 706 | (43.4) | 194 | (43.3) |
| Above average | 3602 | (55.4) | 328 | (42.2) | 827 | (50.9) | 228 | (50.9) |
| Missing/unknown | 53 | (0.8) | 3 | (0.4) | 14 | (0.9) | 2 | (0.4) |
| Self-worth |  |  |  |  |  |  |  |  |
| *“My school is a place where I know I can do well enough to be successful”* |  |  |  |  |  |  |  |  |
| Agree | 6108 | (94.0) | 702 | (90.2) | 1487 | (91.5) | 411 | (91.7) |
| Disagree | 346 | (5.3) | 74 | (9.5) | 124 | (7.6) | 35 | (7.8) |
| Missing/unknown | 47 | (0.7) | 2 | (0.3) | 15 | (0.9) | 2 | (0.5) |
| *“I am a success as a student”* |  |  |  |  |  |  |  |  |
| Agree | 5586 | (85.9) | 608 | (78.2) | 1328 | (81.7) | 367 | (81.9) |
| Disagree | 837 | (12.9) | 158 | (20.3) | 272 | (16.7) | 77 | (17.2) |
| Missing/unknown | 78 | (1.2) | 12 | (1.5) | 26 | (1.6) | 4 | (0.9) |
| *“My school is a place where teachers give me marks I deserve”* |  |  |  |  |  |  |  |  |
| Agree | 5426 | (83.5) | 615 | (79.1) | 1343 | (82.6) | 378 | (84.4) |
| Disagree | 1008 | (15.5) | 158 | (20.3) | 267 | (16.4) | 67 | (15.0) |
| Missing/unknown | 67 | (1.0) | 5 | (0.6) | 16 | (1.0) | 3 | (0.7) |
| Enjoyment of school |  |  |  |  |  |  |  |  |
| *“My school is a place where I get enjoyment from being here”* |  |  |  |  |  |  |  |  |
| Agree | 4829 | (74.3) | 521 | (67.0) | 1142 | (70.2) | 328 | (73.2) |
| Disagree | 1607 | (24.7) | 251 | (32.3) | 473 | (29.1) | 116 | (25.9) |
| Missing/unknown | 65 | (1.0) | 6 | (0.8) | 11 | (0.7) | 4 | (0.9) |
| *“At school I am given the chance to do interesting work”* |  |  |  |  |  |  |  |  |
| Agree | 4885 | (75.1) | 531 | (68.3) | 1196 | (73.6) | 340 | (75.9) |
| Disagree | 1566 | (24.1) | 244 | (31.4) | 418 | (25.7) | 104 | (23.2) |
| Missing/unknown | 50 | (0.8) | 3 | (0.4) | 12 | (0.7) | 4 | (0.9) |
