# Supplementary material for: Long-term recreational exercise patterns in adolescents and young adults: Trajectory predictors and associations with health, mental-health, and educational outcomes
Source: PLoS One. 2024 Mar 21;19(3):e0284660. doi: 10.1371/journal.pone.0284660 (PMC10956783; doi:10.1371/journal.pone.0284660)
Supplement: S5 Table — (DOCX) [file pone.0284660.s016.docx]

# Supplementary table 5. Summary statistics of daily vs less than daily (model 1) trajectory group associations with outcomes at age 25.

| Outcome | Guideline-adherent | | Never guideline | | Guideline drop-out | | Towards guideline | |
| --- | --- | --- | --- | --- | --- | --- | --- | --- |
|  | Freq | (%) | Freq | (%) | Freq | (%) | Freq | (%) |
| Psychological distress (Kessler-6) |  |  |  |  |  |  |  |  |
| Lower risk of mental illness | 543/567 | (95.8) | 1307/1420 | (92.0) | 267/288 | (92.7) | 939/1020 | (92.1) |
| Greater risk of mental illness | 24/567 | (4.2) | 113/1420 | (8.0) | 21/288 | (7.3) | 81/1020 | (7.9) |
| Self-reported general health |  |  |  |  |  |  |  |  |
| Excellent | 169/574 | (29.4) | 184/1432 | (12.9) | 54/291 | (18.6) | 217/1030 | (21.1) |
| Very good | 247/574 | (43.0) | 535/1432 | (37.4) | 110/291 | (37.8) | 367/1030 | (35.6) |
| Good | 115/574 | (20.0) | 503/1432 | (35.5) | 91/291 | (31.3) | 329/1030 | (31.9) |
| Fair | 30/574 | (5.2) | 170/1432 | (11.9) | 31/291 | (10.7) | 96/1030 | (9.3) |
| Poor | 13/574 | (2.3) | 35/1432 | (2.4) | 5/291 | (1.7) | 21/1030 | (2.0) |
| Life satisfaction: happy with life as a whole |  |  |  |  |  |  |  |  |
| Happy | 553/573 | (96.5) | 1350/1416 | (95.3) | 270/287 | (94.1) | 969/1014 | (95.6) |
| Unhappy | 20/573 | (3.5) | 66/1416 | (4.7) | 17/287 | (5.9) | 45/1014 | (4.4) |
| Life satisfaction: happy with the future |  |  |  |  |  |  |  |  |
| Happy | 550/565 | (97.4) | 1315/1381 | (95.2) | 266/280 | (95.0) | 961/995 | (96.6) |
| Unhappy | 15/565 | (2.7) | 66/1381 | (4.8) | 14/280 | (5.0) | 34/995 | (3.4) |
| Completion of high school (Year 12 or Cert II) |  |  |  |  |  |  |  |  |
| Completed Year 12/Cert II | 558/575 | (97.0) | 1391/1438 | (96.7) | 281/292 | (96.2) | 1004/1038 | (96.7) |
| Did not complete | 17/575 | (3.0) | 47/1438 | (3.3) | 11/292 | (3.8) | 34/1038 | (3.3) |
| Completion of any post-school qualification |  |  |  |  |  |  |  |  |
| Yes | 491/575 | (85.4) | 1247/1438 | (86.7) | 240/292 | (82.2) | 871/1038 | (83.9) |
| No | 84/575 | (14.6) | 191/1438 | (13.3) | 52/292 | (17.8) | 167/1038 | (16.1) |
| Participation in the labour force |  |  |  |  |  |  |  |  |
| Employed | 535/552 | (96.9) | 1302/1340 | (97.2) | 256/270 | (94.8) | 945/983 | (96.1) |
| Unemployed | 17/552 | (3.1) | 38/1340 | (2.8) | 14/270 | (5.2) | 38/983 | (3.9) |

Trajectory group-outcome associations were assessed for the subset of participants with outcome data available. Participants missing outcome data were excluded. Psychological distress data were missing for N=6318 participants, self-reported general health data were missing for N=6026 participants, life satisfaction (happy with life as a whole) data were missing for N=6063 participants, life satisfaction (happy with the future) data were missing for N=6132 participants, high-school and post-school qualification data were missing for N=6010 participants, and labour force data were missing for N=6208 participants.
