# Supplementary material for: Long-term recreational exercise patterns in adolescents and young adults: Trajectory predictors and associations with health, mental-health, and educational outcomes
Source: PLoS One. 2024 Mar 21;19(3):e0284660. doi: 10.1371/journal.pone.0284660 (PMC10956783; doi:10.1371/journal.pone.0284660)
Supplement: S6 Table — (DOCX) [file pone.0284660.s017.docx]

# Supplementary table 6. Summary statistics of weekly vs less than weekly (Model 2) trajectory group associations with outcomes at age 25.

| Outcome | Weekly exerciser | | Infrequent exerciser | | Decreasing exerciser | | Increasing exerciser | |
| --- | --- | --- | --- | --- | --- | --- | --- | --- |
|  | Freq | (%) | Freq | (%) | Freq | (%) | Freq | (%) |
| Psychological distress (Kessler-6) |  |  |  |  |  |  |  |  |
| Lower risk of mental illness | 1900/2003 | (94.9) | 231/263 | (87.8) | 641/719 | (89.2) | 284/310 | (91.6) |
| Greater risk of mental illness | 103/2003 | (5.1) | 32/263 | (12.2) | 78/719 | (10.8) | 26/310 | (8.4) |
| Self-reported generalhealth |  |  |  |  |  |  |  |  |
| Excellent | 465/2021 | (23.0) | 27/267 | (10.1) | 96/725 | (13.2) | 36/314 | (11.5) |
| Very good | 813/2021 | (40.2) | 84/267 | (31.5) | 247/725 | (34.1) | 115/314 | (36.6) |
| Good | 570/2021 | (28.2) | 95/267 | (35.6) | 256/725 | (35.3) | 122/314 | (38.9) |
| Fair | 134/2021 | (6.6) | 49/267 | (18.4) | 108/725 | (14.9) | 36/314 | (11.5) |
| Poor | 39/2021 | (1.9) | 12/267 | (4.5) | 18/725 | (2.5) | 5/314 | (1.6) |
| Life satisfaction: happy with life as a whole |  |  |  |  |  |  |  |  |
| Happy | 1938/2007 | (96.6) | 238/263 | (90.5) | 668/709 | (94.2) | 298/311 | (95.8) |
| Unhappy | 69/2007 | (3.4) | 25/263 | (9.5) | 41/709 | (5.8) | 13/311 | (4.2) |
| Life satisfaction: happy with the future |  |  |  |  |  |  |  |  |
| Happy | 1923/1978 | (97.2) | 228/252 | (90.5) | 653/689 | (94.8) | 288/302 | (95.4) |
| Unhappy | 55/1978 | (2.8) | 24/252 | (9.5) | 36/689 | (5.2) | 14/302 | (4.6) |
| Completion of high school (Year 12 or Cert II) |  |  |  |  |  |  |  |  |
| Completed Year 12/Cert II | 1978/2028 | (97.5) | 254/268 | (94.8) | 706/731 | (96.6) | 296/316 | (93.7) |
| Did not complete | 50/2028 | (2.5) | 14/268 | (5.2) | 25/731 | (3.4) | 20/316 | (6.3) |
| Completion of any post-school qualification |  |  |  |  |  |  |  |  |
| Yes | 1741/2028 | (85.8) | 215/268 | (80.2) | 629/731 | (86.1) | 264/316 | (83.5) |
| No | 287/2028 | (14.2) | 53/268 | (19.8) | 102/731 | (13.9) | 52/316 | (16.5) |
| Participation in the labour force |  |  |  |  |  |  |  |  |
| Employed | 1872/1927 | (97.2) | 233/242 | (96.3) | 652/686 | (95.0) | 281/290 | (96.9) |
| Unemployed | 55/1927 | (2.8) | 9/242 | (3.7) | 34/686 | (5.0) | 9/290 | (3.1) |

Trajectory group-outcome associations were assessed for the subset of participants with outcome data available. Participants missing outcome data were excluded. Psychological distress data were missing for N=6318 participants, self-reported general health data were missing for N=6026 participants, life satisfaction (happy with life as a whole) data were missing for N=6063 participants, life satisfaction (happy with the future) data were missing for N=6132 participants, high-school and post-school qualification data were missing for N=6010 participants, and labour force data were missing for N=6208 participants.
